# Triggered Calcium Lightning Programs Cochlear Development

**DOI:** 10.1002/exp2.70205

**Published:** 2026-07-26

**Authors:** Qiang Ma, Hai‐Peng Wang, Yu‐Meng Jiang, Ke Lai, Ming‐Xian Li, Zhen Zhang, Li‐Na Gong, Jing‐Wei Lai, Kai‐Ming Su, Lu‐Yang Wang, Hui Wang, Shan‐Kai Yin, Hao He, Hai‐Bo Shi

**Affiliations:** ^1^ Department of Otorhinolaryngology Head & Neck Surgery Shanghai Sixth People's Hospital Affiliated to Shanghai Jiao Tong University School of Medicine Shanghai China; ^2^ School of Biomedical Engineering Shanghai Jiao Tong University Shanghai China; ^3^ Program in Neurosciences and Mental Health SickKids Research Institute Toronto Canada; ^4^ Department of Physiology University of Toronto Toronto Canada; ^5^ Shanghai Key Laboratory of Sleep Disordered Breathing Shanghai China

**Keywords:** Ano1, cochlea, calcium channel, calcium wave, hearing, inner hair cell, inner supporting cell

## Abstract

Spontaneous calcium (Ca^2+^) waves play a critical role as internal stimuli in the early developing auditory system before the onset of sensory experiences. However, neither the spatiotemporal control of spontaneous Ca^2+^ waves nor their impacts on auditory development are well understood. Here we report that a lateral array of inner supporting cells (ISCs) beneath the inner hair cells displays a spontaneous ultra‐long, ultra‐fast, transient Ca^2+^ flash, termed “Ca^2+^ lightning”, which triggers a series of simultaneous bursts of Ca^2+^ waves in the whole cochlea, before the ear canal opening. The Ca^2+^ lightning margins the cochlear ISCs at different developmental stages that correspondingly exhibit different Ca^2+^ activities. Triggered by an ATP‐induced high‐level Ca^2+^ wave, the Ca^2+^ lightning rapidly propagates laterally into neighboring ISCs through the intricate interplay of the T‐type Ca^2+^ channel, Anoctamin 1 (Ano1) Ca^2+^‐activated Cl^−^ channel and Connexin 26 (Cx26). The genetic deletion of Cav3.2, Ano1, or Cx26 abrogates Ca^2+^ lightning flashes and dysregulates cell development and functions in peripheral cochleae. We propose that cochlear Ca^2+^ lightning flashes orderly with Ca^2+^ waves together orchestrate the spatiotemporal maturation processes of the peripheral auditory system before the onset of hearing.

## Graphical abstract

Summary: Before the onset of hearing, the developing inner ear generates spontaneous calcium signals that are thought to guide maturation. In this study, we discovered a rapid and widespread calcium flash—dubbed “Ca^2^^+^ lightning”—originating from supporting cells beneath the sensory hair cells, which triggers coordinated calcium waves across the entire cochlea through an interplay of specific ion channels and gap junctions. Our findings reveal that this lightning‐like activity is essential for proper cellular development and function in the peripheral auditory system, suggesting that precisely timed internal calcium bursts act as a master clock for preparing the ear before it can respond to sound.

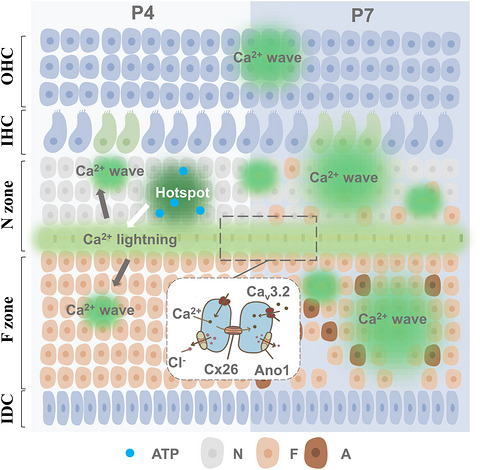

## 1 Introduction

The early development of sensory systems depends on spontaneously generated electrical activity, which is essential to the structural and functional development of the sensory epithelium, mapping of neural circuits, and establishment of sensory functions [1, 2, 3, 4, 5, 6]. Although experience‐dependent refinement of tonotopic maps has been well demonstrated in the auditory cortex, the spontaneous activity before the onset of experience‐driven stimuli is thought to be instructive to the formation of cochlear architecture and projection of neural circuits in peripheral sensory systems. Prominent spontaneous calcium (Ca^2+^) waves, being seemingly random activity, are found in inner supporting cells (ISCs) in the transient structure Kölliker's organ (Ko) before the onset of hearing [7, 8, 9, 10]. The cochlear Ca^2+^ waves are believed to be triggered by the randomly‐released ATP from ISCs. Along with the ATP diffusion that activates P_2_ receptors of ISCs, these ISCs respond to this random event with Ca^2+^ influx, exhibiting a Ca^2+^ wave propagation event. These Ca^2+^ waves enhance and synchronize inner hair cell (IHC) activity [7, 11], a process that is believed to be crucial for coordinating orderly differentiation and functionality of the cochlea. The Ca^2+^ waves that further lead to activation of Ca^2+^‐sensitive Cl^−^ anoctamin channels, and subsequently efflux of Cl^−^, water, and K^+^, in turn depolarize the IHCs, and contribute to excitation of spiral ganglion neurons [12, 13, 14] and establishment of neural connection with IHCs. Immediately after the ear canal opening when external sensory activity takes over to drive the refinement of auditory system and hearing, Ca^2+^ waves disappear. Concurrently, Ko degenerates with significant structural remodeling of the cochlea. However, whether and how local spontaneous Ca^2+^ activities are generated and propagate beyond its territory to globally transduce progressive maturation of the cochlea is still poorly understood.

In this study, we discovered a subset of lateral ISCs close to the IHCs that forms a highly organized array where T‐type Ca^2+^ channels and Anoctamin 1 (Ano1) Ca^2+^‐activated Cl‐ channels, cooperate to coordinate a spontaneously generated, specialized Ca^2+^ wave termed “Ca^2+^ lightning”, which propagates extremely rapidly over long distances much like a lightning flash. These flashes in the cochlea orderly in parallel to the IHC layer to transform local spontaneous high‐level Ca^2+^ waves to global Ca^2+^ activities and regulate the maturation and function of it.

## 2 Results

### 2.1 Ca^2+^ Lightning Triggers Distal Spontaneous Ca^2+^ Waves for Cochlear Development

**FIGURE 1:**
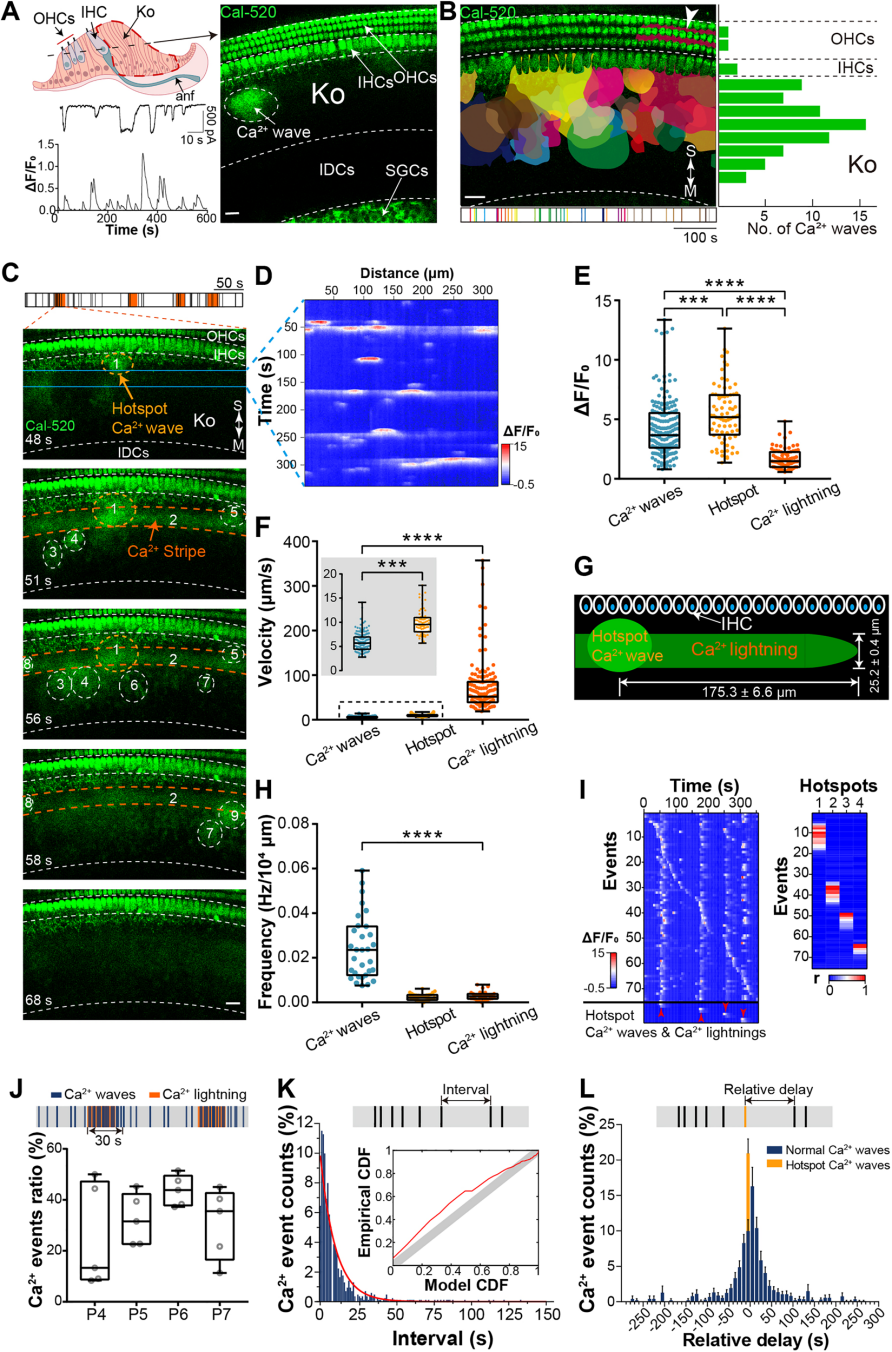
Ca^2+^ lightning as a long‐lasting, ultra‐fast Ca^2+^ flash leads global Ca^2+^ activities. (A) Cochlear spontaneous Ca^2+^ waves indicated by Cal‐520 at 35°C–37°C in serum‐free medium (*n* = 26 cochleae from P4 to P7). Upper left: general structure of a neonatal cochlea (cross section). Lower left: Activity of an ISC indicated by inward currents measured by patch‐clamp (top) or the Ca^2+^ fluorescence signals ΔF/F_0_ by time‐lapse confocal microscopy (bottom). Right: Snapshot of a typical Ca^2+^ wave indicated by raw Cal‐520 fluorescence. ΔF/F_0_: the fluorescence changes over the baseline. anf: Auditory nerve fiber. SGCs: spiral ganglion cells. IDCs: interdental cells. Bar: 20 µm. (B) Overlaid individual Ca^2+^ waves (color‐coded) acquired over a time of 600 s occurred close to IHCs and occasionally spread towards OHCs and Deiter's cells (*n* = 26 cochleae from P4 to P7). Top white arrow: Deiters cells juxtaposed to OHCs. S/M: modiolus (M) and stria vascularis (S) for orientation. Bottom: timeline of spontaneous Ca^2+^ waves, color‐coded according to time of occurrence. Right: spatial distribution of Ca^2+^ waves. Bar: 20 µm. (C) Spontaneous Ca^2+^ waves acquired with confocal microscopy, frame rate 1 frame s^−1^. Ca^2+^ transient likely serving as hotspot for Ca^2+^ lightning (2, dashed lines indicate a Ca^2+^ stripe) and spontaneous Ca^2+^ events (3–9) are labeled in terms of time order (*n* = 24 cochleae from P4 to P7). Top: timeline of spontaneous Ca^2+^ waves (black) and duration of the Ca^2+^ lightning (orange). Bar: 20 µm. (D) The spatiotemporal distribution of Ca^2+^ fluorescence represented by blue lines in C. (E) Relative maximum Ca^2+^ increase and velocity (F) for the different Ca^2+^ phenomena. (*** *p* < 0.001, **** *p* < 0.0001; *n* = 26 cochleae; two‐tailed unpaired *t*‐ test). (G) Average length and width of Ca^2+^ lightning (*n* = 151 from 24 cochleae). (H) Generation frequency of hotspot Ca^2+^ waves, Ca^2+^ lightning, and other spontaneous Ca^2+^ waves (**** *p* < 0.0001; *n* = 26 cochleae from P4 to P7; two‐tailed unpaired *t*‐ test). (I) Heatmap of spontaneous Ca^2+^ waves as a function of time in Ko. Four hotspot Ca^2+^ waves and Ca^2+^ lightning (red‐labeled in the bottom) were observed. Right panel: correlation between Ca^2+^ lightning and other spontaneous Ca^2+^ waves. Bar: 20 µm. "r" is a correlation coefficient. (J) The proportion of Ca^2+^ waves triggered by Ca^2+^ lightning in all Ca^2+^ waves (*n* = 5 cochleae each from P4 to P7). (K) Histogram of time intervals between spontaneous Ca^2+^ waves (*n* = 20 cochleae from P4 to P7). Insert: KS test of temporal generation of spontaneous Ca^2+^ waves. CDF: the Cumulative Distribution Function. (L) Statistics of spontaneous Ca^2+^ waves with different delays relative to hotspot Ca^2+^ wave (*n* = 20 cochleae from P4 to P7). Bar: 20 µm. The cochlea used in Figure 1 were all from rats.

Spontaneous Ca^2+^ activities in freshly isolated rat pup cochleae were observed via time‐lapse confocal imaging of Ca^2+^ fluorescence as Ca^2+^ waves in ISCs in early postnatal development (postnatal day 4–7 (P4‐7)) while simultaneous inward currents were consistently found there (Figure 1A). The cochlear tissue also exhibited periodic optical changes, i.e. the inflation and contraction of cochleae (Supplementary movie 1), consistent with previous reports [7, 11]. Spontaneous Ca^2+^ waves emerged frequently in Ko and spread occasionally to IHCs, inner phalangeal cell, inner border cells, and even Deiters’ cells adjacent to outer hair cells (OHCs) (Figure 1B and supplementary movie 2).

A unique type of Ca^2+^ wave that transiently and anisotropically expanded for several hundreds of microns in a narrow, stripe‐like fashion parallel to IHCs at a high speed, could be distinguished from those Ca^2+^ waves, as highlighted in Figure 1C,D (Ca^2+^ wave 2), termed as “Ca^2+^ lightning”. During the occurrence of Ca^2^^+^ lightning observed via fluorescence imaging, no focal drift was detected in the ISCs in the bright‐field channel (Supplementary Figure 1). The existence of this Ca^2+^ lightning was confirmed in a transgenic CAG‐GCaMP6s mouse model (Supplementary Figure 2A), given the high degree of cross‐species consistency in auditory mechanisms between rats and mice [9, 15, 16]. We verified the consistency of key biomarkers of early cochlear development (Supplementary Figure 2B), cochlear Ca^2+^ waves (Supplementary Figure 2C), and spontaneously active neurons in the cochlear nucleus across the species between rats and mice (Supplementary Figure 2D). In this study, for better surgery, microscopy, and clear demonstration, we used rat pups for the following characterizations of the Ca^2+^ lightning as they provided larger cochlear tissues.

We found that Ca^2+^ lightning always followed a type of Ca^2+^ waves (Ca^2+^ wave 1), the magnitude significantly higher than that of all other spontaneous waves, with a Ca^2+^ fluorescence intensity of 5.46 ± 0.28 ΔF/F_0_, while the propagation speed was also faster than that of normal Ca^2+^ waves, like a Ca^2+^ ignitor (Figure 1E,F). We termed it as a hotspot Ca^2+^ wave and speculated that this triggered Ca^2+^ lightning which in turn gave rise to a series of Ca^2+^ waves within a short time (Ca^2+^ waves 3–9 in Figure 1C).

The magnitude of Ca^2+^ lightning was significantly lower than that of normal Ca^2+^ waves (Figure 1E). The propagation speed of Ca^2+^ lightning was around 70.6 ± 4.6 µm s^−1^, significantly faster than it of all other Ca^2+^ waves, acquired by time‐lapse confocal microscopy at a 2 frame s^−1^ for a field of view of 400 µm × 400 µm (Figure 1F). The temporal correlation of the activation of inward currents/potentials of pairs of double‐patched ISCs was consistent with the rapid propagation characteristic of Ca^2+^ lightning (Supplementary Figure 3). Specifically, the average length and width of the Ca^2+^ lightning stripes were 175.3 ± 6.6 µm and 25.2 ± 0.4 µm, respectively, from the start point of any given hotspot Ca^2+^ wave that gave rise to a stripe (Figure 1G).

Both Ca^2+^ lightning and hotspot Ca^2+^ waves emerged infrequently (Figure 1H). Immediately after the emergence and along with the propagation of Ca^2+^ lightning, a series of Ca^2+^ waves in the whole cochlea were simultaneously generated in the whole cochlea (Figure 1I). Hence the emergence of those spontaneous Ca^2+^ waves was highly correlated with the Ca^2+^ lightning rather than being a random process (Figure 1J). To test this, the distribution of the time interval between all the individual Ca^2+^ waves was shown in the histogram and compared with an exponential distribution using the Kolmogorov‐Smirnov (KS) test (Figure 1K). The interval distribution derived from the 95% confidence interval of the KS plot follows an exponential distribution (insert in Figure 1L). This result suggests the cochlear spontaneous Ca^2+^ waves did not follow a Poisson process. Therefore, the Ca^2+^ waves did not occur randomly; instead, a large part of them consistently emerged in conjunction with the Ca^2+^ lightning, suggesting a strong correlation between some of the spontaneous Ca^2+^ waves and the Ca^2+^ lightning (and hotspot Ca^2+^ waves) (Figure 1L and Supplementary Figure 4).

**FIGURE 2:**
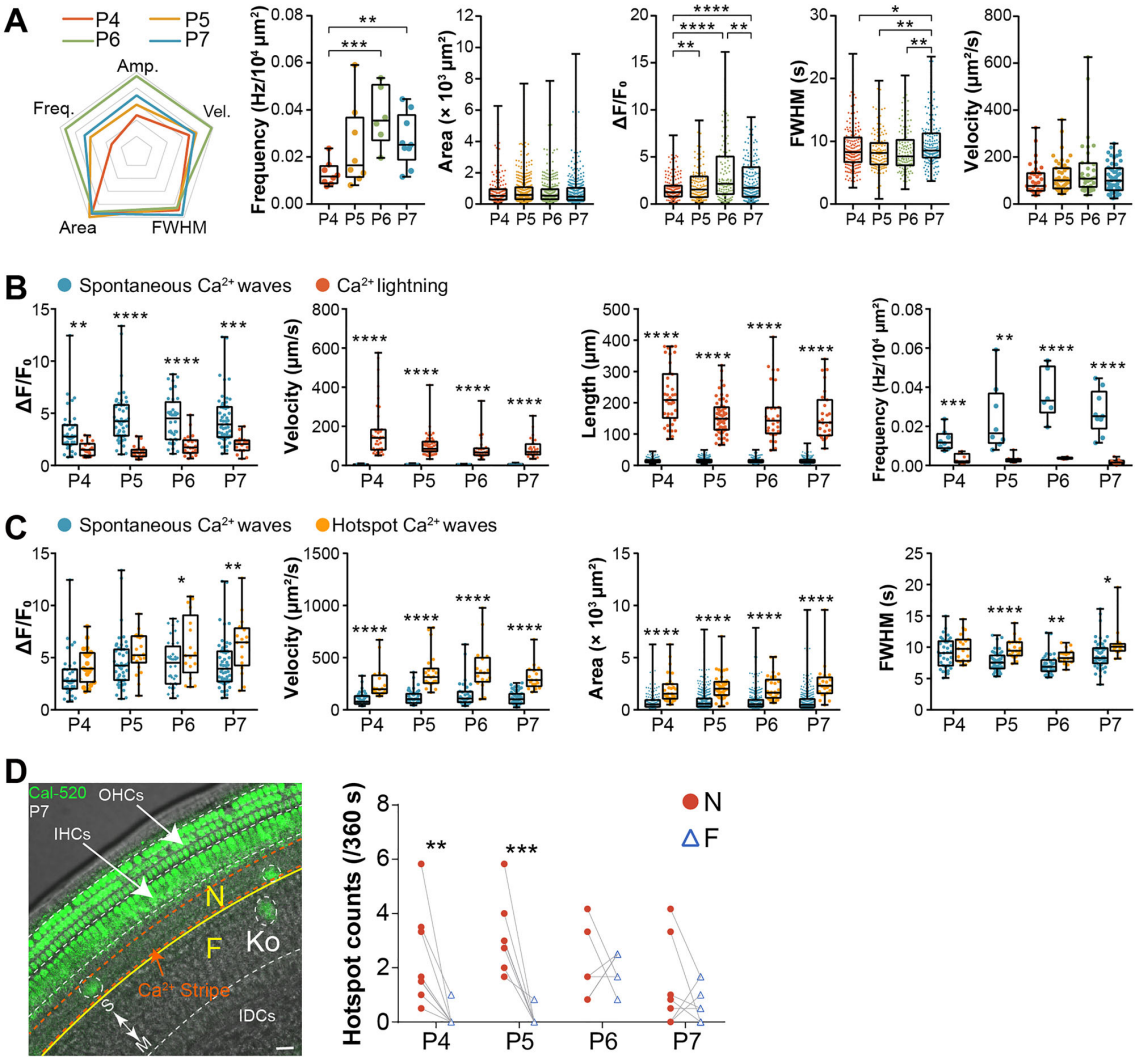
Ca^2+^ lightning is highly related to cochlear maturation. (A) The patterns of spontaneous Ca^2+^ waves vary along with cochlear development from P4 to P7 (*n* = 7, 6, 4, 9 cochleae respectively for P4 to P7). FWHM: full width of the half maximum. Left, the radar chart shows the change in the mean parameter of the Ca^2+^ waves from P4 to P7. (B) The amplitude, propagation velocity, length, and generation frequency of Ca^2+^ lightning (for Fig B, C, *n* = 7, 6, 4, 9 cochleae, respectively, for P4 to P7). (C) The properties of hotspot Ca^2+^ wave compared with other ordinary spontaneous Ca^2+^ waves. (D) Spatiotemporal distribution of spontaneous hotspot Ca^2+^ waves along with the cochlear development (*n* = 7, 6, 4, 9 cochleae, respectively, for P4 to P7; Wilcoxon matched‐pairs signed rank test). Orange dashed lines: Ca^2+^ lightning stripe region. Yellow dashed line: boundary of N and F zones. Bar: 20 µm. The cochlea used in Figure 2 were all from rats. * *p* < 0.05; ** *p* < 0.01; *** *p* < 0.001; **** *p* < 0.0001; two‐tailed unpaired *t*‐ test.

Along with the cochlear development from P4 to P7, the characteristics of spontaneous Ca^2+^ waves were significantly age‐dependent (Figure 2A). The generation of Ca^2+^ waves exhibited more and more frequent along with the age. The Ca^2+^ amplitude, propagation velocity, propagation length, and generation frequency of Ca^2+^ lightning changed accordingly (Figure 2B), but remained the form of low‐Ca^2+^, high‐speed, and ultra‐long stripe. Its trigger, the hotspot Ca^2+^ wave, featured by higher Ca^2+^ level, faster propagation speed, and larger area covered, also changed upon cochlear development (Figure 2C).

We noticed that the generation frequency of hotspot Ca^2+^ waves varied significantly above and below the Ca^2+^ lightning region in the cochlea. As shown in Figure 2D, the region neighboring IHCs, bounded by IHCs and the stripe of Ca^2+^ lightning (orange dashed lines, including the Ca^2+^ lightning stripe), termed the ‘N’ zone, occupied almost all hotspot Ca^2+^ waves in P4‐5 cochleae. On the other side of Ca^2+^ lightning stripe, between it and IDCs (far from IHCs), hotspot Ca^2+^ waves were rarely observed at P4‐5, termed the “F” zone. The generation frequency of spontaneous Ca^2+^ waves also varied significantly in N and F zone, and at P4 and P7, respectively (Supplementary Figure 5). Those results suggest the Ca^2+^ lightning, which was highly correlated with cochlear development, served as a physical and functional boundary that separated ISC subpopulations at distinct maturation stages.

**FIGURE 3:**
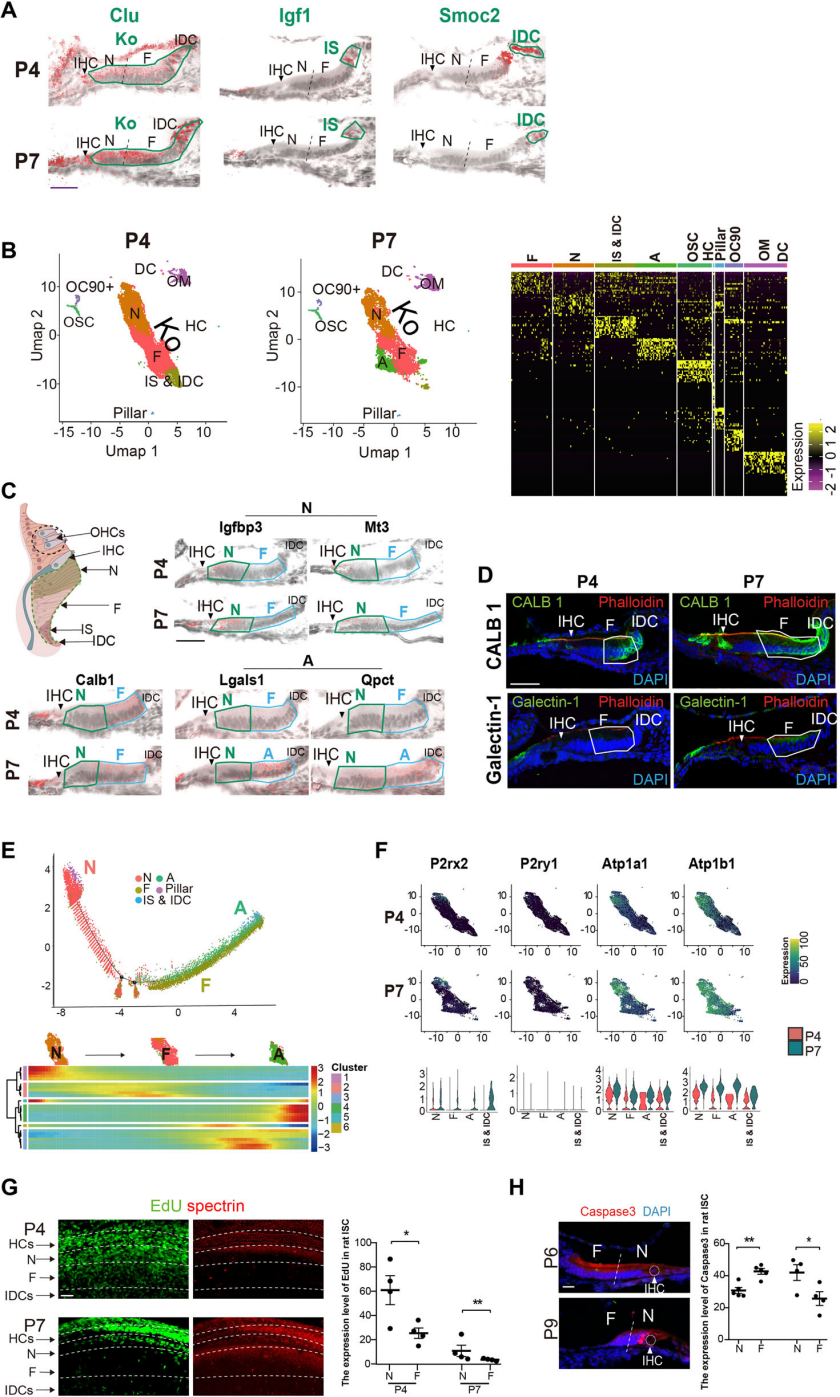
Ca^2+^ lightning coordinates cochlear development. (A) Verification of markers of Ko, IS, and IDC by FISH. Bar: 40 µm. (B) Cell clusters in rat pup cochleae evaluated by UMAP method according to the 10× Genomics data (*n* = 15,027 cells at P4 and 9629 cells at P7 from dissociated Ko from 12 cochleae of 6 rats at each time point). F: F zone; N: N zone; IS: inner sulcus cells; IDC: interdental cells; A: newly‐developed cells in the F zone at P7; OSC: outer sulcus cells; HC: Hair cells; OC90: OC90^+^ cells; OM: Otic Mesenchyme cells; DC: Deiters cells. *Pou4f3* is identified for hair cells (HCs) [17], *Kdr* for Deiters cells [18], *Emid1* and *Pou3f4* for Otic Mesenchyme Cells [19], and a small cluster of cells strongly expressed *Otoconin90*, which is restricted to the cochlear roof [20]. Right panel: Heat map of differentially expressed genes for the 10 identified clusters in ∼24,656 ISCs. (C) FISH images of the gene makers of cluster N, F, and A in Ko at P4 and P7 respectively. Bar: 50 µm. (D) IF microscopy of *Calb1* and *Lgals1* in Ko at P4 and P7 respectively. Bar: 40 µm. (E) Pseudotime analysis of all those cell types. (F) Expression mapping and Violin plots of selected genes including *P2rx2* and *Atp1b1* in cell clusters at P4 and P7. (G) The proliferation of supporting cells in the cochlea of wild‐type rats at postnatal days 4 and 7, along with corresponding statistical analysis, was examined using the EdU labeling method (*n* = 4 cochleae were obtained from both rats at P4 and P7). (H) The apoptosis of supporting cells in the cochlea of wild‐type rats at postnatal days 6 and 9, along with corresponding statistical analysis, was examined using the Caspases3 labeling method (*n* = 5 and 4 cochleae, respectively, for WT rat at P6 and P9). Scale bar: 20 µm. * *p* < 0.05; ** *p* < 0.01; Mann‐Whitney test. The cochlea used in Figure 3 were all from rats.

Therefore, we postulate that Ca^2+^ lightning stripe marks the boundary where the ISCs belong to different developmental stages. In this regard, we determined the developmental features of the cochlear cells by single‐cell RNA sequencing of rat pup cochleae at P4 and P7 via 10× Genomics (Supplementary Figure 6). The cell clusters of ISCs, inner sulcus (IS) cells and IDCs from the sequencing data could be identified by marker genes including *Clu*, *Smoc2* and *Igf1* (Supplementary Figure 6), consistent with the fluorescence in situ hybridization (FISH) of them in fresh cochleae, respectively (Figure 3A). Those results confirmed the validity of cell clustering and their markers analyzed by this single‐cell RNA sequencing algorithm.

By this method, the subtypes of ISCs in the N and F zones at different developmental stages could be explicitly distinguished by their transcripts at P4 and P7 respectively (Figure 3B and Supplementary Figure 7). By FISH imaging in the cochlear sections at P4 and P7 respectively, we found cluster N and F exactly colocalized with N and F zones in cochleae, margined by the Ca^2+^ lightning (Figure 3B,C). Those results were further verified by immunofluorescence microscopy of the marker proteins (Figure 3D). We found a new cell cluster (labeled as A, the additional cluster for apoptosis) that was separated by the transcriptional changes in the F zone at P7 (Figure 3B,C), featured by markers including *Thrb* and *Lgals1* (*Galectin‐1*) that probably worked for the final apoptosis and degeneration of ISCs for the formation of inner sulcus [21, 22]. According to the pseudotime analysis, the N, F, and A clusters emerge in an orderly manner along the developing trajectory of the cochlea, indicating ISCs develop from the N state to the F state and finally the A state, consistent with the degeneration fate of ISCs (Figure 3E and Supplementary Figure 8). Specifically, from P4 to P7, ISCs in those clusters exhibited increased expression of P_2_ receptors and ATPase, suggesting more and active hotspot Ca^2+^ waves were initiated (Figure 3F), consistent with the more frequent Ca^2+^ waves observed at P7 (Figure 2A). We verified the cell fates predicted by the pseudotime analysis based on the EdU labeling and Caspase‐3 immunofluorescence staining of cochlea at P4 ‐ P9, respectively. The proliferation and apoptosis of ISCs in the cochlea of rats on P4‐P9 were generally consistent with the cell fates in the N and F/A regions (Figure 3G,H). Hence, the Ca^2+^ lightning, together with the Ca^2+^ waves, orchestrates the location and activity of ISCs from N to F (and A) zones in an age‐dependent manner, exactly corresponding to the developmental stages of different cell clusters.

### 2.2 Ca^2+^ Lightning Is Triggered by ATP and Pulsated by Synergistic Actions of T‐Type Ca^2+^ Channel, Ano1 and Connexin 26

**FIGURE 4:**
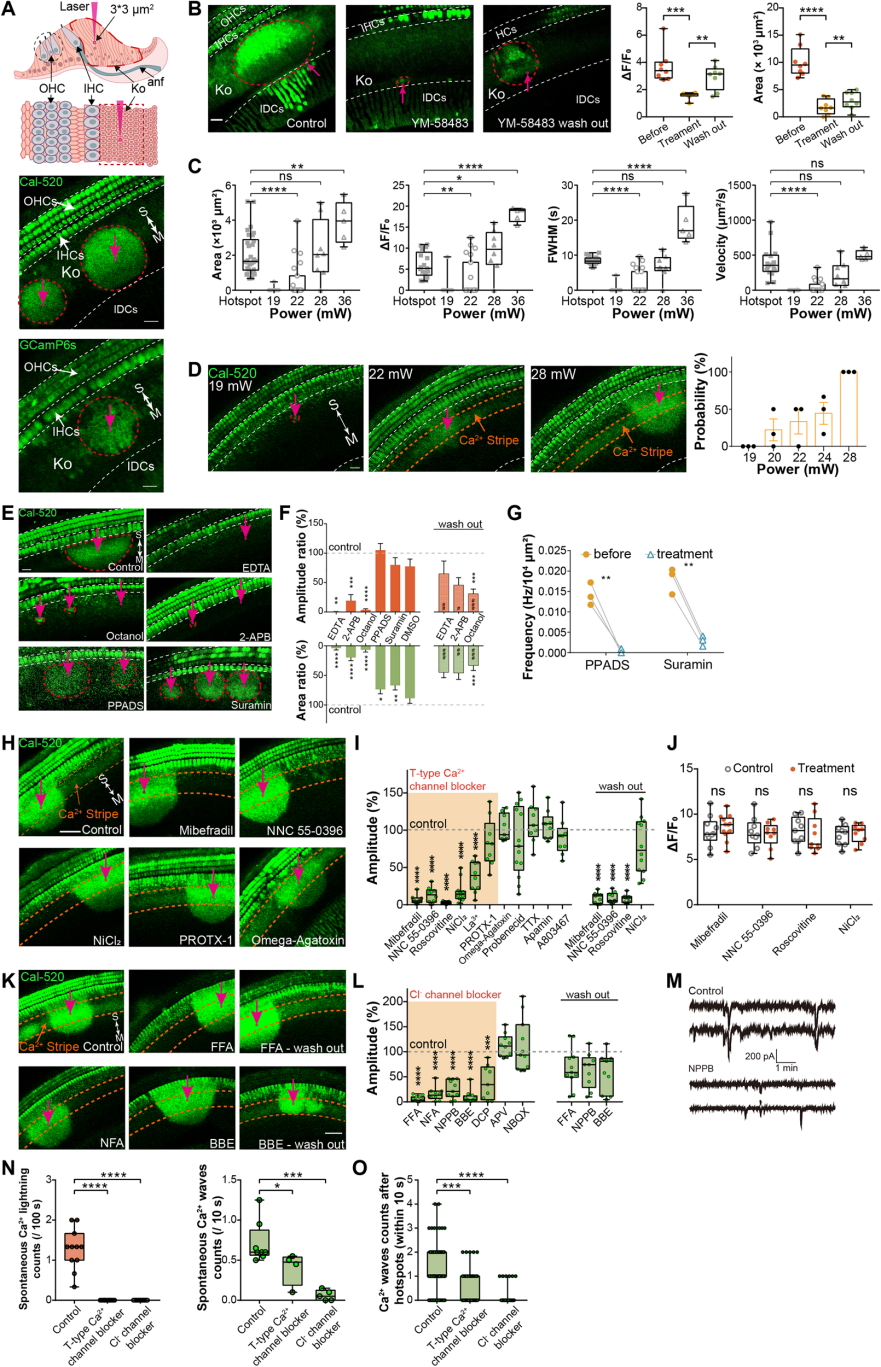
Ion channels involved in hotspot Ca^2+^ waves and Ca^2+^ lightning. (A) A tightly‐focused femtosecond laser excited Ca^2+^ waves from a single randomly‐selected targeted ISC, indicated by Cal‐520 and GCamP6s in Ko respectively. Bar: 20 µm. (B) The Ca^2+^ waves could not be excited by laser in the presence of the SOC‐specific blocker YM‐58483 (200 µM, *n* = 4 cochleae from P6). (C) Characteristics of laser‐induced Ca^2+^ waves by different laser powers compared with the spontaneous hotspot Ca^2+^ waves (*n* ≥ 5 experiments in 3 cochleae at P6 for each laser power, *n* = 30 experiments in 4 cochleae at P6 for the hotspot Ca^2+^ waves). (D) Dependence of Ca^2+^ lightning on laser power. Left: examples of Ca^2+^ phenomena induced by a focused laser beam with the laser power indicated. Right: probability of Ca^2+^ lightning on laser power (*n* ≥ 3 trails in 3 rat cochleae per power level). Arrows: site of laser activation. Bar: 20 µm. (E) The laser‐excited hotspot Ca^2+^ waves in cochleae at P4 and P7 in the presence of reagents: 1) ethylenedinitrilotetraacetic acid (EDTA) [24]; 2) octanol [25]; 3) 2‐Aminoethyl diphenylborinate (2‐APB) [26]; 4) pyridoxal‐phosphate‐6‐azophenyl‐2', 4'‐disulphonate (PPADS) [27]; 5) suramin [28] and 6) dimethyl sulfoxide (DMSO). Bar: 20 µm. (F) The amplitude and propagation area of laser‐excited hotspot Ca^2+^ waves in Ko treated with different reagents (EDTA 3.3 mM; octanol 4.2 mM; 2‐APB 800 µM; PPADS 333 µM; suramin 1.5 mM and control: DMSO 1%) and then washed out (*n* ≥ 5 trials in each cochlea and *n* = 3 cochleae in per group). (G) Frequency of spontaneous hotspot Ca^2+^ waves before and after PPADS or suramin treatment (*n* = 3 cochleae per group; ** *p* < 0.01; Wilcoxon matched‐pairs signed rank test). (H) The fluorescent images of Ca^2+^ lightning excited by the laser‐induced hotspot Ca^2+^ waves in the presence of (1) Mibefradil [29]; (2) NNC 55–0396 [30]; (3) NiCl_2_ [31]; (4) ProTx‐I [32] and (5) omega‐Agatoxin TK [33]. Bar: 50 µm. (I) The quantified amplitude of Ca^2+^ lightning in the presence of (1) Mibefradil; (2) NNC 55–0396; (3) Roscovitine [34]; (4) NiCl_2_; (5) La^3+^ [35]; (6) ProTx‐I; (7) omega‐Agatoxin TK; (8) Probenecid; (9) tetrodotoxin (TTX) [36]; (10) Apamin [37] and (11) A803467 [38], and with reagents (1‐4) wash‐out (Mibefradil 50 µM; NNC 55–0396 50 µM; Roscovitine 250 µM; NiCl_2_ 2.5 mM; La^3+^;400 µM; ProTx‐I 2 µM; omega‐Agatoxin TK 30 nM; Probenecid 400 µM; TTX 5 µM; Apamin 6 µM and A803467 400 nM). (J) Hotspot Ca^2+^ waves could still emerge in the presence of T‐type Ca^2+^ channels inhibitors. (K) The fluorescent images of Ca^2+^ lightning excited by the laser‐induced hotspot Ca^2+^ waves in the presence of (1) niflumic acid (NFA); (2) flufenamic acid (FFA); (3) benzbromarone (BBE), and with FFA and BBE wash out. Bar: 50 µm. (L) The quantified amplitude of Ca^2+^ lightning in the presence of: (1) FFA; (2) NFA; (3) 5‐Nitro‐2‐(3‐phenylpropylamino) benzoic acid (NPPB) [39]; (4) BBE; (5) dichlorophen (DCP) [9, 40]; (6) D‐2‐Amino‐5‐phosphonovaleric acid (APV) [41]; and (7) 2,3‐dihydroxy‐6‐nitro‐7‐sulphamoyl‐benzo (F)‐quinoxaline (NBQX) [42], and with FFA, NPPB and BBE wash‐out, respectively (FFA 300 µM; NFA 200 µM; NPPB 450 µM; BBE 100 µM; DCP 100 µM; APV 500 µM and NBQX 50 µM), *n* = 5 for Probenecidand). (M) Electrophysiological signals by double patch clamp recording from two ISCs in the presence of NPPB. (N) The counts of spontaneous Ca^2+^ lightning and Ca^2+^ waves (within 10 s) in the presence of T‐type Ca^2+^ channel blockers and Cl^−^ channel blockers respectively. (O) The Ca^2+^ waves related to the Ca^2+^ lightning in the presence of T‐type Ca^2+^ channel blockers and Cl^−^ channel blockers. The cochlea used in Figure 4 were all from rats (*n* ≥ 3 trials in each cochlea and *n* ≥ 3 cochleae in per group). Arrows: laser focus. * *p* < 0.05; ** *p* < 0.01; **** *p* < 0.0001; Mann‐Whitney test.

We then clarified the mechanism of Ca^2+^ lightning generation. Since the Ca^2+^ lightning flashes were relatively rare, we developed an optical method to stimulate an individual targeted ISC and initiate a local Ca^2+^ wave with high spatiotemporal precision [23], to generate controllable hotspot Ca^2+^ waves (Figure 4A). The mechanism is the same as previously reported, that the photostimulation to cochlear ISCs excited Ca^2+^ rise through the activation of the store‐operated Ca^2+^ (SOC) channel (Figure 4B). The laser excited distinct patterns of Ca^2+^ waves at different powers (Figure 4C and Supplementary Figure 9, movie 3). We found the Ca^2+^ waves activated at 28 mW reliably recapitulated the characteristics of the hotspot Ca^2+^ waves at P4 and P7. In following experiments, we used 28 mW as the power to trigger hotspot Ca^2+^ waves in cochlear tissues. This laser‐induced hotspot Ca^2+^ wave then activated Ca^2+^ lightning with high fidelity and the downstream spontaneous Ca^2+^ waves (Figure 4D). Note that Ca^2+^ lightning and other Ca^2+^ waves could be stably triggered by laser stimulation. Instead, ATP treatment to cochlear tissues usually induced uncontrollable activation of Ca^2+^ lightning and waves with random delays and abnormally prolonged durations of them (Supplementary Figure 10).

To verify that Ca^2+^ lightning was triggered by the hotspot Ca^2+^ wave, we utilized this optical method to excite hotspot Ca^2+^ waves in fresh cochleae in the presence of reagents to respectively deplete Ca^2+^, inhibit gap junctions, IP_3_R, and P_2_ purinoceptors (Figure 4E,F, and Supplementary movie 4). Localized Ca^2+^ rise in an individual ISC could be still excited by laser in the presence of octanol, but it was only limited to the targeted cells, and no Ca^2+^ wave was formed. The laser‐induced hotspot Ca^2+^ waves were largely abolished in the groups treated with EDTA, octanol, and 2‐APB respectively. The laser (at 28 mW) could excite hotspot Ca^2+^ waves in the PPADS and suramin treated cochleae (Figure 4E,F), but PPADS and suramin significantly suppressed spontaneous hotspot Ca^2+^ waves (Figure 4G). Taken together, these results suggest hotspot Ca^2+^ waves were initiated from individual ISCs where ATP induced local high‐level Ca^2+^ rise and propagated through gap junction, causing Ca^2+^ release from intracellular stores and Ca^2+^ influx in the neighboring ISCs. This is consistent with the high‐level Ca^2+^ observed in hotspot Ca^2+^ waves (Figure 2C) and the high‐level expression of P_2_ receptors and ATPase found by RNA sequencing (Figure 3F). If the laser‐induced hotspot Ca^2+^ waves were inhibited, Ca^2+^ lightning or those simultaneous Ca^2+^ waves triggered by it were greatly suppressed. In the presence of PPADS and suramin, the laser‐induced hotspot Ca^2+^ waves still had high‐level Ca^2+^ and could trigger Ca^2+^ lightning (Figure 4E,F), further suggesting Ca^2+^ lightning was triggered by the high‐level Ca^2+^ of hotspot Ca^2+^ waves.

We then clarified the generation mechanism of Ca^2+^ lightning by screening ion channels of ISCs. By using series of broad‐spectrum and highly specific inhibitors/blockers of cation channels and Ca^2+^ channels, we found hotspot Ca^2+^ waves largely failed to trigger Ca^2+^ lightning in the presence of specific inhibitors of T‐type Ca^2+^ channels (Mibefradil [29], NNC55‐0396 [30], La^3+^ [35], Roscovitine [34], and NiCl_2_ [31]) (Figure 4H,I). Here Mibefradil and La^3^^+^ block T‐type Ca^2+^ channels but may have off‐target effects. NNC 55–0396 is a highly selective T‐type Ca^2+^ channel blocker [43], and ProTx‐I, a potent and selective CaV3.1 channel blocker (with roughly 160‐fold more potency than through Cav3.2 channels. Omega‐Agatoxin TK, a specific inhibitor of CaV2.1 (P/Q‐type) Ca^2+^ channel [33], did not influence Ca^2+^ lightning (Figure 4I). Neither voltage‐activated Na^+^ nor SK2 K^+^ channels were involved in Ca^2+^ lightning either because addition of TTX [36], A803467 [38], or Apamin [37] had no effect. Furthermore, hotspot Ca^2+^ waves could still emerge in the presence of T‐type Ca^2+^ channels inhibitors (Figure 4J), although the Ca^2+^ lightning and downstream Ca^2+^ waves were suppressed by them. These results suggested the T‐type Ca^2+^ channels mediated the Ca^2+^ influx to drive spontaneous electrical activity in ISCs underlying Ca^2+^ lightning, which also further confirmed the Ca^2+^ lightning was triggered by hotspot Ca^2+^ waves and simultaneously triggered a series of Ca^2+^ waves in a large region of the cochlea.

We then examined the hypothesis that the Ca^2+^ lightning was generated by a pacemaker and thus further screened if any channels of anions or receptors work as the pacemaker cation carrier for depolarization. The broad‐spectrum blockers of Cl^−^ channels (NFA, FFA, NPPB [39]), and specific blockers (BBE [40], DCP [9]) of the Ca^2+^ activated Cl^−^ channel Ano1 (TMEM16A), suppressed Ca^2+^ lightning significantly (Figure 4K,L). The inhibition of Ano1 channel significantly suppressed the active electrical activity in ISCs in Ko (Figure 4M). We therefore suggest that Ano1, a Ca^2+^‐activated Cl^−^ channel, serves to depolarize the membrane potential and together with T‐type Ca^2+^ channels contribute to the generation and spread of Ca^2+^ lightning. In those experiments, if the Ca^2+^ lightning was inhibited, some spontaneous Ca^2+^ waves still remained while some downstream Ca^2+^ waves failed to initiate (Figure 4N,O). Therefore, Ca^2+^ lightening is locally sparked by an ATP‐induced hotspot Ca^2+^ wave and then propagates in the whole cochlea to activate global Ca^2+^ waves along the basal‐lateral axis in cochlea.

**FIGURE 5:**
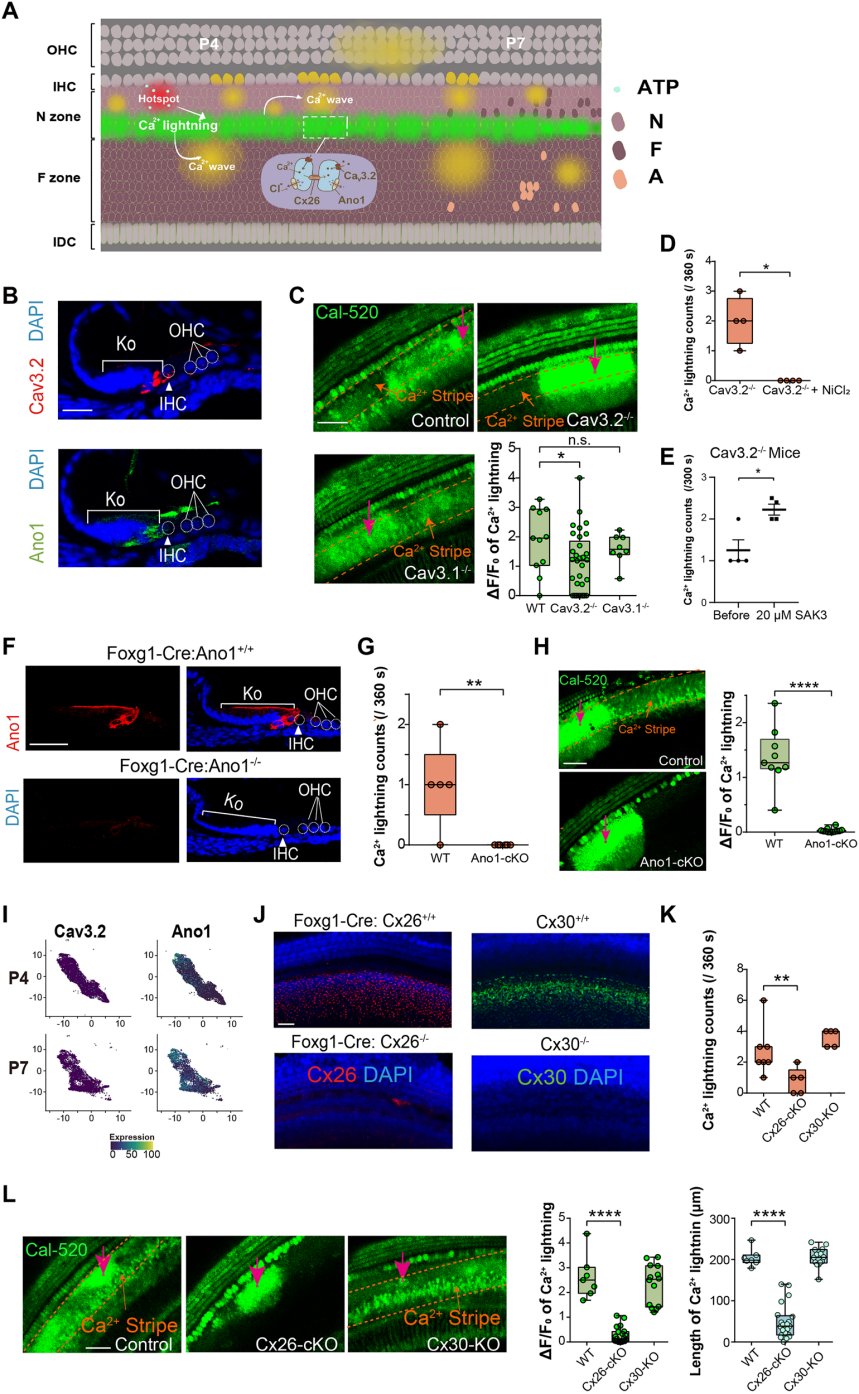
Ca^2+^ lightning is formed through cochlear Cav3.2, Ano1, and gap junction Connexin 26. (A) Summary of the initiation and regulation mechanism of Ca^2+^ lightning that programs cochlear development. (B) Immunofluorescence images of Cav3.2 and Ano1 in cochlear cross sections at P7 from wild type mouse. Bar: 20 µm. (C) The amplitude of Ca^2+^ lightning excited by laser‐induced hotspot Ca^2+^ waves in Cav3.2‐KO and Cav3.1‐KO mice (*n* = 4, 2, 10 cochleae, respectively, for WT, Cav3.1‐KO and Cav3.2‐KO mice). (D) The Spontaneous Ca^2+^ lightning in Cav3.2‐KO mice in the presence of NiCl_2_ (*n* = 4 cochleae). (E) SAK3 (20 µM) facilitates the recovery of spontaneous Ca^2+^ lightning in the basilar membrane of the cochlea in Cav3.2‐KO. (n = 4 cochleae obtained from Cav3.2‐KO mice and wild‐type rats). (F) Immunofluorescence images of Ano1 of cochleae from Ano1‐cKO mice. Bar: 50 µm. (G) Spontaneous Ca^2+^ lightning in Ano1‐cKO cochleae (for both Figure 5G and H *n* = 3, 4 cochleae respectively for WT and Ano1‐cKO;). Bar: 50 µm. (H) The amplitude of Ca^2+^ lightning excited by laser‐induced hotspot Ca^2+^ waves in Ano1‐cKO mice. Bar: 50 µm. (I) The expression of Cav3.2 and Ano1 in ISCs at P4 and P7, respectively. (J) The immunofluorescence images of Cx26 and Cx30 in cochleae of Cx26‐cKO and Cx30‐KO mice, respectively. Bar: 20 µm. (K) The spontaneous Ca^2+^ lightning in cochleae of Cx26‐cKO and Cx30‐KO mice, respectively. (L) The amplitude and length of Ca^2+^ lightning excited by laser‐induced hotspot Ca^2+^ waves in cochleae of Cx26‐cKO and Cx30‐KO mice (For both Figure 5K and L, *n* = 5, 7, 5 cochleae, respectively, for WT, Cx26‐cKO and Cx30‐KO). The cochlear in Figure 5B and 5I were from rats, in Figure 5C‐H and J‐L from mouse models. * *p* < 0.05; ** *p* < 0.01; **** *p* < 0.0001; Mann‐Whitney test.

Although ISCs are unexcitable cells, based on these results, we propose that the intricate interplay between the T‐type Ca^2+^ channels and Ano1 facilitates the generation of the Ca^2+^ lightning while Connexin 26 (Cx26) gap junctions transmit electric fluctuation to the neighboring ISCs so as to empower the Ca^2+^ lightning (Figure 5A). We performed immunofluorescence microscopy of cochlear sections to localize them in cochleae. As shown in Figure 5B, both Cav3.2 (a representative of T‐type Ca^2+^ channels) and Ano1 are expressed in ISCs close to IHCs, consistent with the location of Ca^2+^ lightning.

To validate the role of T‐type Ca^2+^ channels and Ano1, we constructed Cav3.2‐KO mice and a conditional Ano1‐cKO mouse (Foxg1‐Cre: Ano1^−/−^) model (Supplementary Figure 12). In Cav3.2‐KO cochleae, the Ca^2+^ lightning was suppressed but not completely (Figure 5C). The incomplete elimination of Ca^2+^ lightning was probably due to the compensatory responses by other T‐type Ca^2+^ channels like Cav3.1 that was over expressed in Cav3.2‐KO mice (Supplementary Figure 12C). We found the Ca^2+^ lightning was not influenced in Cav3.1‐KO mice, but totally inhibited in Cav3.2‐KO cochleae in the presence of NiCl_2_, the inhibitor of T‐type channels (Figure 5C,D). Given the short duration of the early cochlear developmental stage (before P12) that is not enough to perform viral injections into the cochlea and achieve sufficient Cav3.2 expression, we opted instead for SAK3, a potent T‐type voltage‐gated Ca^2+^ channels enhancer that has been shown to augment the currents of Cav3.1 and Cav3.3 isoforms [44] to temporarily rescue Cav3.2 function. Consistently, SAK3 (20 µM) treatment of the Cav3.2‐KO cochleae at P4 demonstrated a significant recovery of spontaneous Ca^2+^ lightning (Figure 5E).

Consistently, spontaneous Ca^2+^ lightning in Ano1‐cKO mice disappeared (Figure 5F,G). The laser stimulation could only trigger a local Ca^2+^ hotspot wave but failed to trigger any Ca^2+^ lightning (Figure 5H). These results were consistent with the pharmacological screening, indicating T‐type Ca^2+^ channels and Ano1 depolarized ISCs for generating Ca^2+^ lightning despite ISCs lacking intrinsic excitability. Moreover, the expression of Cav3.2 and Ano1 increased from P4 to P7 (Figure 5I), indicating the ISCs are more electrically coupled, consistent with the more frequent Ca^2+^ waves observed along age (Figure 2A).

The fast intercellular Ca^2+^ propagation of Ca^2+^ lightning should be supported by gap junctions between ISCs. We then examined the major gap junction proteins Cx26 and Connexin 30 (Cx30) by constructing Cx26‐cKO (Foxg1‐Cre: Cx26^−/−^) and Cx30‐KO (Cx30^−/−^) mouse models (Figure 5J). The spontaneous Ca^2+^ lightning could rarely be found in cochleae of Cx26‐cKO mice, but seen normally in Cx30‐KO mice (Figure 5K). Both the amplitude and propagation length of the Ca^2+^ lightning triggered by the laser‐induced hotspot Ca^2+^ wave in the Cx26‐cKO mice were significantly less than those of wild‐type mice but showed no difference in Cx30‐KO mice (Figure 5L). Hence, Cx26 is necessary for mediating the intercellular Ca^2+^ lightning propagation.

### 2.3 Ca^2+^ Lightning Contributes to Cochlear Maturation

**FIGURE 6:**
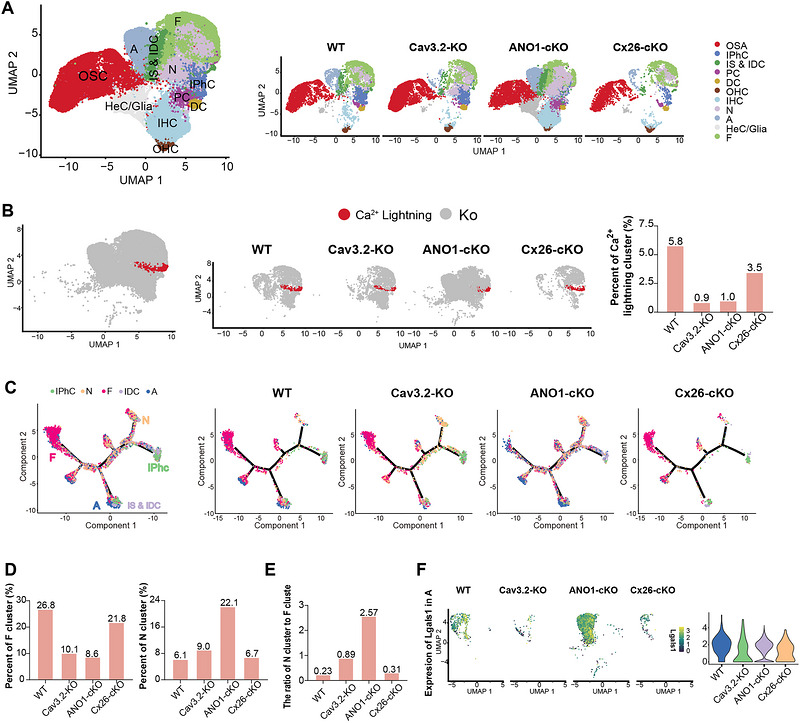
Ca^2+^ lightning defect affects cochlear development. (A) Cell clusters in cochleae from Cav3.2‐KO (*n* = 10,613 cells), Ano1‐cKO (*n* = 28,935 cells), Cx26‐cKO (*n* = 4,689 cells), and WT (*n* = 7493 cells) mouse evaluated by UMAP (*n* = 10, 8, 10, 12 cochleae respectively for Cav3.2‐KO, Ano1‐cKO, Cx26‐cKO, and WT mouse at P7). (B) The cells working for Ca^2+^ lightning ISCs. (C) The developmental trajectories of each cell subpopulations in those models and WT mice. (D) The cell ratio of ISC clusters in each mouse model and WT mice. (E) The ratio of N cluster to F cluster in each mouse model and WT mice. (F) The expression of Lgals1 and Cluster A in each mouse model. The cochlea used in Figure 6 were all from mice.

To explore the possibility that the Ca^2+^ lightning was essential to cochlear development and hearing formation, we constructed Cav3.2‐KO, Ano1‐cKO, and Cx26‐cKO mouse models to partially verify the critical role of Ca^2+^ lightning in cochlear development. Due to the experimental difficulty of constructing rat models, we used mouse models here, which might introduce some genetic differences. Fortunately, the cell clusters acquired by the markers from rats were generally consistent in mouse models (Figure 6A). The subtypes of cochlear cells in these knockout models presented distinct clusters compared with the WT group. The subset of the Ca^2+^ lightning ISCs could then be identified according to the loss of it in those models (Figure 6B and Supplementary Figure 11). Given the Ca^2+^ lightning was partially inhibited, we found defective Ca^2+^ lightning greatly affected the development trajectory of the cochlea (Figure 6C and Supplementary Figure 13). Specifically, Cluster F decreased but N increased in Cav3.2‐KO and Ano1‐cKO mouse models (Figure 6D). The N and F cell clusters were far different from it under the normal developmental conditions (Figure 6E). Cluster A, the mature ISCs in the later stage of cochlear development (Figure 3C), were significantly diminished in all three models, indicating a delay of cochlear development in those models (Figure 6F).

**FIGURE 7:**
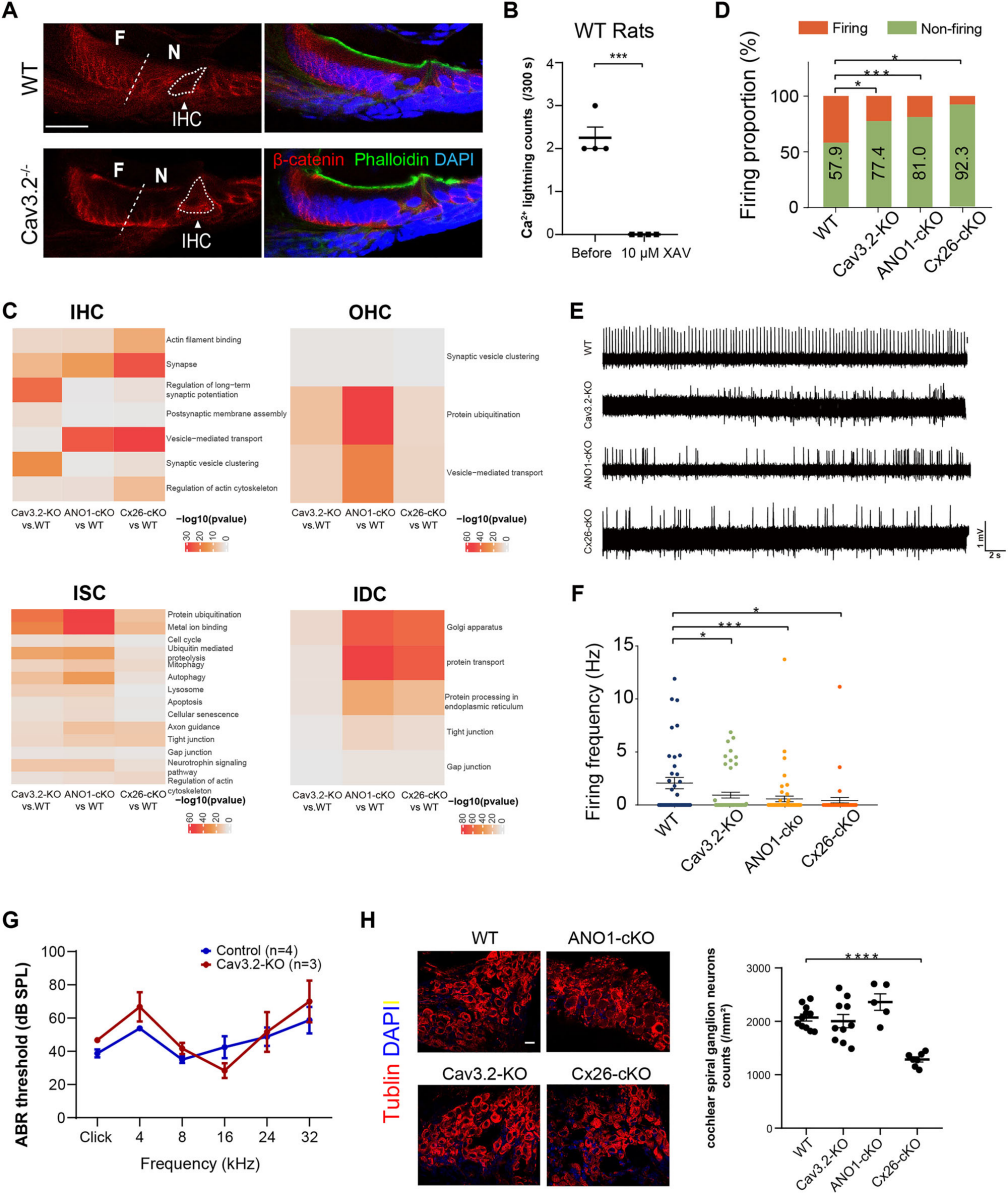
Ca^2+^ lightning affects cochlear maturation and functions. (A) Immunofluorescence images of β‐catenin in cochlear cross sections at P7. Bar: 50 µm. (B) The Wnt signaling pathway‐specific inhibitor XAV939 (10 µM) completely suppressed spontaneous Ca^2+^ transients in wild‐type rats (n = 4 cochleae). (C) KEGG and GO analysis of OHCs, IHCs, ISCs, and IDCs in those Ca^2+^ lightning defect mice. (D) The proportion of spontaneous firing neurons in the cochlear nuclei of those Ca^2+^ lightning defect mice. (E) The spontaneous firing of neurons in cochlear nuclei from the brains of those mouse models. (F) The frequency of spontaneous firing in the firing neurons in cochlear nuclei from those mouse models. (For both Figure 7D and F, *n* = 53, 58, 39, 38 cochlear nucleus neurons, respectively, for Cav3.2‐KO, Ano1‐cKO, Cx26‐cKO, and WT mouse at P10) * *p* < 0.05. *** *p* < 0.001 by Mann–Whitney U test and chi‐squared test (χ2). (G) ABR testing on Cav3.2‐KO mice at P30. (H) SGN in Tubb3‐labeled P7 to P14 mouse models and their statistical analysis. (*n =* 5,4, 4,4 cochleae, respectively, for WT, Cav3.2‐KO, Ano1‐cKO and Cx26‐cKO at P4 and P7).Scale bar: 20 µm. post‐hoc test (Tukey test) following one‐way ANOVA, Mann‐Whitney test, **** *p* < 0.0001. The cochlea used in Figure 7 were all from mice.

The Ca^2+^ lightning regulates local Wnt signals for cochlear development in ISCs [45]. In WT mice, Wnt signaling is activated in N/F clusters but suppressed in IHCs. Conversely, Cav3.2‐KO models show downregulated Wnt in N/F clusters with aberrant activation in IHCs (Figure 7A). When the Wnt was inhibited by XAV, a specific inhibitor of the Wnt, spontaneous Ca^2+^ lightning was then suppressed (Figure 7B). The molecular markers in cochlear cells from Cav3.2‐KO, Ano1‐cKO, and Cx26‐KO mouse models revealed that the vesicle transport and neuronal connection in hair cells, development and maturation of ISCs, and secretion functions (for the formation of the tectorial membrane) of IDCs were significantly affected (Figure 7C). By electrophysiological measurements on the brain tissues from those Ca^2+^ lightning‐defect mouse models, we found that the proportion of spontaneously firing neurons and the firing events in those active neurons in the cochlear nuclei in those mutant mice were both significantly smaller than that in WT control (Figure 7D–F). Auditory brainstem response (ABR) testing on Cav3.2‐knockout (KO) mice at P30 indicates that the absence of Cav3.2 (Ca^2+^ lightning) does not impair peripheral hearing function (Figure 7G). We performed immunofluorescence microscopy to examine SGNs in the cochlea using tublin staining across the transgenic mouse models. The morphology and density of SGNs in Cav3.2‐KO and Ano1‐KO mice were unaffected by the loss of Ca^2+^ lightning (Figure 7H). These findings are consistent with the ABR results, indicating preserved hearing function in these mouse models. Our observations align with previous studies reporting no hearing loss in Ano1‐KO mice [46, 47]. In contrast, Cx26‐KO mice exhibited significant SGN degeneration, likely because of the disruption of gap junction‐mediated intercellular communication, which compromises cochlear structural integrity. Due to the great difficulty of specific conditional knockout of Cav3.2, Ano1, or Cx26 in ISCs in cochleae, we demonstrate the single‐time injection of the Ano1 blocker BBE into cochleae of rats could significantly suppress their hearing function at P30 (Supplementary Figure 14). Hence, these data indicate Ca^2+^ lightning plays an essential role in programming cochlear development. Perturbing key molecular substrates underlying Ca^2+^ lightning slows down the functional maturation of both peripheral and central auditory system.

## 3 Discussion

Our study unraveled a new form of cochlear Ca^2+^ wave, ‘Ca^2+^ lightning’ flashes that travel laterally for up to several hundred micrometers at an ultra‐fast speed along an array of ISCs close to IHCs. Ca^2+^ lightning is locally sparked by an ATP‐induced hotspot Ca^2+^ wave and then propagates in the whole cochlea to activate global Ca^2+^ waves along the basal‐lateral axis in the cochlea, as an amplifier of spontaneous activity to expand Ca^2+^ activity to the entire cochlea, stimulating inner and outer hair cells, not only synchronizing their development, but also coordinating orderly differentiation and functionality of them and facilitating the maturation of cochlear architecture and function prior to hearing onset. Although ISCs are unexcitable cells, we found that an intricate interplay between the T‐type Ca^2+^ channels and Ano1 supports the Ca^2+^ lightning while Cx26 gap junctions transmit electric fluctuation to the neighboring ISCs so as to empower the Ca^2+^ lightning (Figure 5A). The fate of ISCs in Ko is apoptosis for the programmed degeneration of Kölliker's organ [48] and the functional maturation of Corti's organ, consistent with the ISCs developmental trajectory. During regression of Ko, apoptosis and proliferation occur simultaneously (N and A clusters) [49]. Aberrant intracellular Ca^2+^ signaling mediated by Cx26 may serve as a key regulatory mechanism underlying dysregulation of the autophagy‐apoptosis signaling axis, though this requires further investigation [50]. In this regard, we define Ca^2+^ lightning as a fast, long Ca^2+^ wave propagating along ISCs expressing T‐type Ca^2+^ channel, Ano1, and Cx26, which works as an “amplifier” of the spontaneous activity that enhances intrinsic Ca^2+^ activities to promote cochlear development and the maturation of cochlear structure and function prior to hearing onset. It also serves as a synchronizing event and a spatial boundary marker for ISCs differentiation.

The development of neural circuits relies on spontaneous electrical activity that occurs during immature stages of development. In the auditory system, patterned firing activity has been observed in immature spiral ganglion and brain‐stem neurons and is likely to depend on cochlear IHC action potentials [51]. Prior to the onset of vision, the retina exhibits spontaneous activities termed retinal waves for approximately 2 weeks after birth, disappearing around the time of eye‐opening. Ca^2+^ waves in retina glial cells exhibit propagation characteristics highly analogous to the Ca^2+^ lightning observed in the cochlea [1, 52]. The development of sensory systems and even the brain also requires such internal Ca^2+^ stimulations to trigger cell differentiation and neuronal projections. Therefore, we propose such similar Ca^2+^ wave activities should also be found in the early developmental stages of other sensory systems.

We found *P_2_rx2*, a cation channel gated by extracellular ATP, expressed highly in N zone at P4, consistent with the spatiotemporal frequency of hotspot Ca^2+^ wave generation (Figure 3F). *Atp1a1* and *Atp1b1*, enzymes that catalyze the hydrolysis of ATP, showed a matched distribution with *P_2_rx2*. The cells from cluster N are highly active in metabolism and sensitive to stimuli. Cluster F and A cells are much more quiescent but the magnitude of their Ca^2+^ waves is higher and longer. Strikingly, Ca^2+^ waves on both sides of the Ca^2+^ lightning are strongly associated with it. Although we constructed Cav3.2‐KO, Ano1‐cKO, and Cx26‐cKO mouse models, however, we did not knock out those genes specifically in the ISCs for Ca^2+^ lightning due to the extreme experimental difficulty. Hence, even though we observed the inhibition of Ca^2+^ lightning arrests the cochlear development and hearing functions in these models, the knockout of those genes in the entire cochlea might also contribute to the phenomena we described in Figures 6 and 7. Our results thus provide critical mechanistic insights into previous sporadic observations showing ATP, Ano1, and Cx26 are essential to hearing function [7, 9, 47, 53].

The other family of Ca^2+^‐activated Cl^−^ channel in the cochlea, bestrophins (*BEST*1‐3), exhibits quite low expression in cochleae and thus does not contribute to Ca^2+^ lightning (Supplementary Figure 15). Both BEST1 and Ano1 are activated by intracellular Ca^2+^; however, voltage‐dependent activation and outward rectification are only observed for Ano1 [54]. Therefore, Ano1 that exhibits a higher sensitivity to Ca^2^^+^, enabling it to respond effectively to spontaneous Ca^2^^+^ activities in the cochlea, facilitates intrinsic activities for early sensory organ development better than BEST1.

The Ca^2+^ lightning orchestrates the location and activity of ISCs from N to F zones in an age‐dependent manner, exactly corresponding to the developmental stages of different cell clusters. Our findings showing Ca^2+^ lightening regulates the N, F, and A clusters highlight its pivot role in governing the developing trajectory of cochleae, in line with a recent work that reported the emergence of distinct subpopulations of cochlear cells according to direct single‐cell RNA sequencing of mouse cochleae at P1 and P7 [55]. Notably, Ca^2+^ lightning is deeply involved with Wnt, which has been demonstrated to participate in proliferation, organ of Corti formation, planar cell polarity (PCP), and convergent extension movements of the cochlear duct in the developing inner ear [45, 56, 57]. Functionally, Ca^2+^ lightning may be associated with congenital hearing loss, particularly in the context of ANO1, Cx26 and Cav3.2‐related pathologies. In this study, the absence of Ca^2+^ lightning in the Ko did not affect cochlear structure or ABR thresholds in mice (Figure 7G,H), but significantly impaired neuronal maturation within the cochlear nucleus (Figure 7D–F). These findings suggest that peripheral cochlear Ca^2+^ activity plays a regulatory role in the development and maturation of the central auditory system. Recent experimental evidence supports our hypothesis. In Ano1‐deficient mice, although cell survival or acoustic thresholds remain intact, the patterned burst discharge of central auditory neurons is disrupted, accompanied by reduced burst firing activity and diminished frequency selectivity [46]. Furthermore, functional refinement of the projection from the medial nucleus of the trapezoid body (MNTB) to the lateral superior olive (LSO) is compromised, indicating deficits in synaptic circuit maturation [47]. Cav3.2 has been implicated in both age‐related and noise‐induced hearing loss [58]. Such forms of sensorineural hearing loss may be mechanistically linked to disrupted Ca^2+^ lightning in the Ko, leading to aberrant central auditory connectivity and impaired circuit refinement. Taken together, we conclude that Ca^2+^ lightning is a novel form of signaling that programs the maturation of the cochlear and/or degeneration of Ko before ear canal opening, ultimately contributing to the establishment of structural and functional connectivity between the peripheral and central auditory systems.

## 4 Methods

### 4.1 Preparation of Fresh Cochleae

Collagen gel was prepared to provide an appropriate environment for the cochlear explants to grow. The preparation protocols of collagen gel and serum‐free medium are described in Ding D et al. [59]. Briefly, rat tail collagen (Type 1, BD Biosciences; Bedford, MA) was gently mixed with 10× Eagle's Basal Medium (BME; B9638, Sigma; St. Louis, MO) and 2% sodium carbonate at a ratio of 9:1:1 prior to use. Next, 15 µL of collagen gel was placed in the center of a 35‐mm culture dish (Falcon 353001, Northbrook, IL) for 30 min to let the collagen solidify. Then 1 mL of serum‐free medium was applied to cover the collagen gel. The serum‐free medium used in the present study contained 2 g of bovine serum albumin (BSA; A‐4919, Sigma; St. Louis, MO), 2 mL of Serum‐Free Supplement (I‐1884, Sigma; St. Louis, MO), 4.8 mL of 20% glucose (G‐2020, Sigma; St. Louis, MO), 1 mL of Ampicillin Sodium Solution (B540722, Sangon Biotech), 2 mL of 200 mM glutamine (G‐6392, Sigma; St. Louis, MO), and 190.8 mL of 1× basal medium eagle (BME) (B‐1522, Sigma; St. Louis, MO).

After quickly removing the temporal bone, cochlear explants were dissected out and placed in ice‐cold Hank's Balanced Salt Solution (HBBS). The membranous labyrinth was exposed by removing the bony shell of the inner ear. After dissecting out the cochlear basilar membrane, the tectorial membrane was gently pulled out and the explants were placed on the previously‐prepared collagen gel and serum‐free medium. The explants were incubated at 37°C in 5% CO_2_ for 15 min to allow the explants to grow on the surface of collagen gel. More medium could be added if necessary.

### 4.2 Fluorescence Imaging of Intracellular Free Ca^2+^ Concentration Changes in Ko

For Ca^2+^ imaging, explants were incubated with 5 µM Cal‐520/AM (21130, AAT Bioquest) for 40 min at 37°C while being protected from ambient light. After staining with Cal‐520/AM, explants were washed and the buffer was replaced with serum‐free medium.

For a genetically encoded indicator of intracellular free Ca^2+^ concentration [Ca^2+^] i, a transgenic mouse model with GCaMP6s was established by the following protocol. GCaMP6s knockin mice were acquired using the CRISPR/Cas9 system. First, one sgRNA‐targeting the near sequence of the inserted site H11 was constructed and transcribed in vitro. The donor vector with the inserted fragment CAG‐LSL‐GCaMP6s‐PolyA was designed and constructed in vitro. Then Cas9 mRNA, sgRNA H11‐S2 (5′‐ CTGAGCCAACAGTGGTAGTA‐3′), and donor were co‐injected into zygotes. Thereafter, the zygotes were transferred into the oviduct of pseudopregnant Institute of Cancer Research (ICR) females at 0.5 dpc. F0 mice were born after 19∼21 days of transplantation. All the offspring of ICR females (F0 mice) were identified by polymerase chain reaction (PCR) and sequencing of DNA from their tails. Finally, crossing positive F0 mice with wildtype mice was used to build up heterozygous mice.

To activate GCaMP6s in GER cells, we obtained a B6/JGpt‐*H11^emCin(CAG‐Cre)^*/Gpt transgenic mouse line that expresses *Cre* in GER cells. Crossing positive GCaMP6s knockin mice with positive B6/JGpt‐*H11^emCin(CAG‐Cre)^*/Gpt mice were used to build up heterozygous mice.

B6/JGpt‐*H11^emCin(CAG‐Cre)^*/Gpt mice were genotyped using primers recognizing the CAG sequence (5′‐CCTGCTGTCCATTCCTTATTCCATA‐3′and 5′‐ ATATCCCCTTGTTCCCTTTCTGC ‐3′) that generated a 337‐bp band showing the presence of the CAG allele. GCaMP6s knockin mice were genotyped using primers recognizing the GCaMP6s sequence (5′‐TCAGCGTTCAGACTCCTCAGAATGT‐3′and 5′‐ TCAATGGAAAGTCCCTATTGGCGT ‐3′) that generated a 1701‐bp band. B6/JGpt‐*H11^emCin(CAG‐Cre)^*/Gpt. GCaMP6s knockin mice of both sexes were purchased from Gempharmatech. Co., Ltd.

### 4.3 Materials

The following reagents were used at their corresponding concentrations (or the protocol from the manufacturers): octanol, 4.2 mM; 2‐aminoethyl diphenylborinate (2‐APB), 800 µM; pyridoxal‐phosphate‐6‐azophenyl‐2', 4'‐disulphonate (PPADS), 333 µM; flufenamic acid (FFA), 300 µM; niflumic acid (NFA), 200 µM; LaCl_3_, 400 µM; Glutamine, 200 mM; NaCl, 124 mM; KCl, 5 mM; CaCl_2_, 2.4 mM; KH_2_PO_4_, 1.2 mM; NaH2PO4, 1.3 mM;NaHCO_3_, 24 mM; EGTA,0.6 mM; HEPES, 10 mM; Sodium creatine phosphate dibasic tetrahydrate, 10 mM; Na_3_GTP, 0.3 mM; Na_2_ATP, 3 mM; Potassium gluconate, 130 mM; Thermolysin, 0.5 mg mL^−1^; Dnase, 5 g mL^−1^; DNase I,10 µg mL^−1^; 1×BME; 10×BME; MgCl_2_; BSA; Serum‐Free Supplement; glucose; dimethyl sulfoxide (DMSO); DAPI; NiCl_2_, 2.5 mM from Sigma; Trypsin, 0.25%; FBS, 5%; 1× red blood cell lysis solution; Trypan blue, 0.4%; DPBS; ViewRNATM ISH Cell Assay Kit from Thermo Fisher; dichlorophen (DCP), 100 µM; Apamin, 6 µM; A803467, 400 nM; D‐2‐Amino‐5‐phosphonovaleric acid (APV) 500 µM; 2,3‐dihydroxy‐6‐nitro‐7‐sulphamoyl‐benzo (F)‐quinoxaline (NBQX), 50 µM; Probenecid, 400 µM; Mibefradil dihydrochloride, 50 µM; NNC 55–0396, 50 µM; Roscovitine, 250 µM; 5‐Nitro‐2‐(3‐phenylpropylamino) benzoic acid (NPPB); SAK3, 20 µM; XAV, 10 µM; benzbromarone (BBE) from MCE; ethylenedinitrilotetraacetic acid (EDTA), 3.3 mM; HBBS; Ampicillin Sodium Solution; 1×PBS; Triton X‐100 from Sangon Biotech; Rabbit anti‐Calbindin, 1:200; Phalloidin, 1:2000; Rabbit anti‐beta Catenin 1:200; ProTx‐I, 2 µM; omega‐Agatoxin TK, 30 Nm; Rabbit anti‐ beta Tubulin 1:200 from Abcam; Rabbit anti‐Connexin 30, 1:200; Mouse anti‐Connexin 26, 1:200 from Invitrogen; Alexa Fluor 488, 1:500; Alexa Fluor 555, 1:500 from Life; Suramin, 1.5 mM from Calbiochem; TTX, 5 µM from Alomone labs; Cal‐520/AM, 5 µM from AAT Bioquest; Rabbit anti‐Galectin‐1, 1:200 from Novus; Tissue storage solution from Biotechnology; Sucrose from Biofroxx; OCT from TissueTek; Rat tail collagen from BD Biosciences; Veterinary tissue adhesive from Millpledge Ltd; Rabbit anti‐Connexin 26, 1:1000 from Proteintech; Rabbit anti‐Ano1, 1:1000; Rabbit anti‐Cacna1g, 1:200 from Affinity Biosciences; Rabbit anti‐SOX2, 1:1000 from Abclonal; Cleaved Caspase‐3 (Asp175) Antibody, 1:200 from Cst; EdU, 10 µM from Beyotime; αII‐spectrin, 1:200 from BioLegend.

### 4.4 Mouse Models

Gjb6 KO mice (NCBI ID 14623), Gjb2loxP/loxP mice (NCBI ID 14619), and Cav3.2 KO mice (NCBI ID 58226) were obtained from Shanghai Model Organisms Center, Inc; Ano1 f/f mice (NCBI ID 101771), Foxg1‐P2A‐iCre mice (NCBI ID 15228) were obtained from Gempharmatech. Co., Ltd; Cav3.1 KO mice (NCBI ID 12291) were obtained from Cyagen Biosciences.

Ano1 and Gjb2 conditional null mutants (Ano1 cKO, Gjb2 cKO) were generated by crossing Ano1 f/f mice and Gjb2f/f mice with Foxg1‐P2A‐iCre mice, respectively.

All experimental procedures regarding the care and use of animals were reviewed and approved by the blinded information for review (Approval Number: 2019‐0246). All efforts were made to minimize possible pain and discomfort of the animals during the experimental procedures.

### 4.5 Ca^2+^ Imaging and Analysis

The fresh cochleae were prepared in 35‐mm dishes and observed by confocal microscopy using an upright two‐photon confocal microscope (A1 MP+, Nikon). A water‐immersion objective (Nikon, 40×, 0.8 N.A.) was used to acquire wide‐field and fluorescent images. The green fluorescence of Cal‐520 and GCaMP6s was excited by a 488‐nm laser at around 0.2 mW. To simultaneously observe Ca^2+^ transients in the whole Ko, the image plane was determined by ISCs in the acute cochlear samples that lay flat at the dish bottom to minimize out‐of‐focus Ca^2+^ events. Confocal images were usually acquired for 2.2 µs pixel^−1^ with 512 × 512 pixels (1 s frame^−1^) and further processed by ImageJ for cropping, rotation, and contrast adjustment. The microscopy period of each sample was limited to 2 h to maintain a healthy cochlear status. All microscopy trials were performed at room temperature (∼22°C).

### 4.6 Photostimulation

A femtosecond laser (tunable from 690 to 1040 nm, ∼100 fs, 80 MHz, MaiTai DeepSee, Spectra‐Physics) on the two‐photon confocal microscope (A1 MP+, Nikon), which shared the same light path of two‐photon microscopy, was used for photostimulation to excite the Ca^2+^ transients in Ko. The randomly‐selected target ISCs were stimulated by the femtosecond laser by defining a two‐photon microscopy region, usually a rectangular area ∼ 3 × 3 µm^2^ in the ISCs. The small region was scanned for a one‐time, short duration stimulation (500 ms) by the focused femtosecond laser to stimulate Ca^2+^ waves propagating from the target ISC. At the sample, the femtosecond laser power was 15–40 mW at 800 nm. If necessary, the photostimulation process could be inserted into any pre‐defined confocal microscopy sequences as a single two‐photon microscopy frame for continuous observation. The femtosecond laser was blocked by an optical shutter during confocal imaging.

### 4.7 Patch Clamping for Electrophysiology

#### 4.7.1 Electrophysiological Recording of the ISCs

Middle turns of SD rat pups aged P4 or P7 were isolated in ice‐cold artificial cerebrospinal fluid (aCSF) containing (in mM): 124 NaCl, 5 KCl, 2.4 CaCl_2_, 1 MgCl_2_, 1.2 KH_2_PO_4_, 24 NaHCO_3_ and 10 glucose, saturated with 95% O_2_/5% CO_2_. The stria vascularis and tectorial membrane were gently removed, and the cellular organization of the organ of Corti was left intact. Cochlear turns were held in place by a circular glass coverslip with a fine insect pin. Tissues were continually superfused with aCSF at 22–24 °C, and used within 3 hours of the dissection.

The stripe region was identified in the acutely isolated cochlear turn with a 60× water immersion objective attached to an upright microscope (Examiner.A1, Zeiss, Germany). As mentioned above, the stripe region was located in Ko with 15–30 µm distances from IHCs. Patch pipettes were pulled from borosilicate capillary glass by using a vertical pipette puller (PC‐10; Narishige, Japan) and had tip resistances of 2–4 MΩ when filled with internal solution containing (in mM): 130 K‐gluconate, 5 KCl, 0.6 ethylene glycol tetraacetic acid (EGTA), 10 4‐(2hydroxyethyl)‐1‐piperazineethanesulfonic acid (HEPES), 4 MgCl_2_, 10 sodium creatine phosphate dibasic tetrahydrate, 3 Na_2_ATP and 0.3 Na_3_GTP (adjusted to pH 7.3 with KOH, 290 mOsm L^−1^). All experiments were performed at room temperature (23–27°C). Electrodes were advanced into the tissue using minimal positive pressure to prevent disruption of the close spatial relationship between ISCs. All recordings were made with the whole cell patch‐clamp technique using a patch‐clamp amplifier (MultiClamp‐700B, Axon, America), and all data were sampled at 10–12 kHz and filtered at 1–3 kHz using a Dell computer equipped with pClamp6 software.

Spontaneous inward currents were recorded from ISCs in the stripe region, under voltage‐clamp mode with a pipette potential of −80 mV. To clarify whether synchronous spontaneous currents existed between ISCs in the stripe region, double‐patch recordings were performed under voltage‐clamp mode with the holding potentials at ‐80 mV. Synchronous spontaneous currents could be observed in all ISCs from stripe regions with the distances of 3–53 µm between ISCs.

The pharmacological agents used in the experiment are listed below: NPPB, 50 µM, BBE, 10 µM. Pharmacological reagents were dissolved in a CSF and applied through a square‐tube gravity perfusion system. Spontaneous currents were recorded for 10 min in the absence of chemicals, and then recorded for an additional 10 min after applying the compound for a 10 min equilibration period.

#### 4.7.2 Electrophysiological Recording of the Cochlea Nucleus Neurons

Cav3.2‐KO, Ano1‐cKO, Cx26‐cKO, and WT mice and rats were deeply anesthetized with isoflurane, and the brain was rapidly removed and cut transversely at 300 µm using a vibratome (VT1200S, Leica Microsystems). Slices were transferred to aCSF containing (in mM): 119 NaCl, 2.5 KCl, 2.5 CaCl_2_, 1.3 MgCl_2_, 1.3 NaH_2_PO_4_, 26 NaHCO_3_, and 20 glucose, at 35°C for 30 min and then allowed to recover for 30 min at room temperature before recordings.

Cell‐attach patch clamp recordings were made from cochlear nucleus neurons in a recording chamber mounted on a fixed‐stage upright microscope (Zeiss). Patch electrodes (4‐6 MΩ) were made from 1.5 mm borosilicate glass (World Precision Instruments, USA). The intracellular solution contained (in mM): 140 K‐gluconate, 10 HEPES, 2 MgCl_2_, 1 CaCl_2_, 0.5 EGTA, 2 MgATP, (290‐300 mOsm, pH 7.3, 4–5 MΩ). Current‐clamp recordings were made by an EPC‐10 patch‐clamp amplifier (HEKA). Data were acquired at 10–20 kHz and filtered at 1–3 kHz using a computer equipped with the Pulse 6.0 software (HEKA, Lambrecht). Cells were recorded at membrane potential (I = 0).

### 4.8 Isolation of Cochlear Cells

The cochlear basilar membrane was dissected from P4 / P7 SD rats and Cav3.2‐KO, Ano1‐cKO, Cx26‐cKO, and WT mice at P7 and incubated in serum‐free medium containing 0.5 mg mL^−1^ thermolysin and 5 g mL^−1^ DNase for 30 min at 37°C. After the junction between the epithelium and mesenchymal tissue of the cochlea became loosened, a clean cochlear epithelial sheet (CES) mainly composed of the greater epithelial ridge (GER), inner hair cells (IHCs), outer hair cells (OHCs) and lesser epithelial ridge (LER) could easily be separated from the underlying basal membrane and mesenchyme using fine dissecting forceps. CES from mice were transferred to tissue storage solution (OE Biotech Co., Ltd, Shanghai) for storage with a 20 µL pipette.

Ko isolation. In order to obtain intact and clean GER sheets, all the inner cells were kept while trimming away all lateral areas, including IHCs, which indicate the boundary of those GER sheets. Any connective tissue cells at the edge of the GER area were removed. Finally, such collected GER from rat was transferred to tissue storage solution (Biotechnology Corporation, Shanghai) for storage with a 20 µL pipette.

### 4.9 Gene Sequencing

#### 4.9.1 Tissue Dissociation and Cell Purification

CES and GER were transported in a sterile culture dish with 10 mL of 1× dulbecco's phosphate‐buffered saline (DPBS; Thermo Fisher, Cat. no. 14190144) on ice to remove the residual tissue storage solution and then minced on ice. We used dissociation enzyme 0.25% trypsin (Thermo Fisher, Cat. no. 25200‐072) and 10 µg mL^−1^ DNase I (Sigma, Cat. no. 11284932001) dissolved in phosphate buffered saline (PBS) with 5% fetal bovine serum (FBS; Thermo Fisher, Cat. no. SV30087.02) to digest the tissues. CES and GER were dissociated at 37°C with a shaking speed of 50 r.p.m. for about 40 min. We repeatedly collected the dissociated cells at interval of 20 min to increase cell yield and viability. Cell suspensions were filtered using a 40‐µm nylon cell strainer. Red blood cells were removed by 1× red blood cell lysis solution (Thermo Fisher, Cat. no. 00‐4333‐57). Dissociated cells were washed with 1× DPBS containing 2% FBS. Cells were stained with 0.4% trypan blue (Thermo Fisher, Cat. no. 14190144) to check the viability on the Countess II Automated Cell Counter (Thermo Fisher).

#### 4.9.2 10× Library Preparation and Sequencing

Beads with unique molecular identifiers (UMIs) and cell barcodes were loaded close to saturation, so that each cell was paired with a bead in a gel beads‐in‐emulsion (GEM). After exposure to cell lysis buffer, polyadenylated RNA molecules hybridized to the beads. Beads were retrieved into a single tube for reverse transcription. On cDNA synthesis, each cDNA molecule was tagged on the 5’end (that is, the 3’end of a messenger RNA transcript) with UMI and cell tag. Briefly, 10× beads that were then subject to second‐strand cDNA synthesis, adaptor ligation, and universal amplification. Sequencing libraries were prepared using randomly interrupted whole‐transcriptome amplification products to enrich the 3’ end of the transcripts linked with the cell barcode and UMI. All the remaining procedures, including the library construction, were performed according to the standard manufacturer's protocol (CG000206 Rev D). Sequencing libraries were quantified using a High Sensitivity DNA Chip (Agilent) on a Bioanalyzer 2100 and the Qubit High Sensitivity DNA Assay (Thermo Fisher Scientific). The libraries were sequenced on NovaSeq6000(Illumina) using 2 × 150 chemistry.

#### 4.9.3 Data Analysis

Raw sequencing reads of the cDNA library were mapped to the reference genome by 10× Genomics Cell Ranger pipeline (https://support.10xgenomics.com/single‐cell‐gene‐expression/software/downloads/latest) using default parameters. The R package Seurat [60] was utilized for subsequent analysis. Firstly, raw gene expression matrices were read and converted into Seurat objects. We filtered low quality cells that have unique feature counts less than 500 or over 4000. Cells with more than 20% mitochondrial genes were also excluded from the analysis. The gene expression matrix was then normalized according to the total cellular unique molecular identifier (UMI) count. Top 2000 features were selected as highly variable genes for further clustering analysis. In order to reduce dimensionality, principal component analysis (PCA) was performed based on the highly variable genes after scaling the data with respect to UMI counts. Afterwards, the first 50 principal components were chosen for downstream dimensionality reduction and clustering. The transcriptional markers of each cluster were calculated using the Find All Markers function with the Wilcoxon test under the following criteria: log2 fold change > 0.25 and min. pct > 0.25. Top markers of each cluster were then selected to perform a heatmap plot. Differentially expressed genes (DEGs) were selected using the function FindMarkers (test. use = presto). *p* value < 0.05 and log2 fold change > 1.5 was set as the threshold for significantly differential expression. GO enrichment and KEGG pathway enrichment analysis of DEGs were respectively performed using R (version 4.0.3) based on the hypergeometric distribution.

#### 4.9.4 Single‐Cell Pseudotime Analysis

After cell type annotation, we performed single‐cell pseudotime analysis for each cell type separately using Monocle2 [61] with the DDR‐tree reduction method (http://cole‐trapnell‐lab.github.io/monocle‐release/).Single‐cell pseudotime analysis was performed with default parameters to eliminate batch effects except minor differences. Briefly, the raw gene expression matrix was converted into a Monocle object. During feature selection, the intersection of top marker genes of Seurat clusters and differential genes between samples was set as the ordering genes for downstream pseudotime analysis. Trajectory plots and heatmaps were utilized to show pseudotime results.

Slingshot analysis. Slingshot [62] could provide the lineage structure and pseudotime variable inferences for cells along each lineage. The slingshot analysis was performed by the slingshot R package (version 1.8.0). Initially, the dimensionally reduced Seurat object was transformed into a SingleCellExperiment object by as Single Cell Experiment function. The N cluster was selected as a starting cluster for the trajectory inference. Then, the FitGAM function in the tradeSeq package [63] (version 1.4.0) was used to fit the nonlinear function between gene expression and pseudotime using a negative binomial generalized additive model (NB‐GAM). By using the associationTest function, the top 100 genes whose expression levels significantly differed from pseudotime were chosen to create a heatmap.

### 4.10 Immunofluorescence Microscopy

Immunostaining was performed on whole‐mount preparations of basilar membrane and cryostat sections of cochleae. Rats at P4 and P7 were decapitated. The cochleae were quickly removed to acquire fresh explants. For surface preparations, basilar membranes were carefully dissected out and then fixed in 4% paraformaldehyde for 1 h at room temperature. For cryosections, cochleae were fixed in 4% paraformaldehyde for 1 h at room temperature and then decalcified in 10% EDTA at room temperature for 2–6 h until the cochlea became soft. Afterwards, the samples were immersed with 15% and 30% sucrose in PBS at 4°C for 1 h, respectively, and finally embedded and frozen in OCT (TissueTek). In this study, the cryosections were 10 µm thick.

Primary antibodies used include rabbit anti‐Galectin‐1 (1:200, NBP2‐75499, Novus), rabbit anti‐Calbindin (1:200, ab108404, Abcam), rabbit anti‐Connexin 30 (1:200; 700258, Invitrogen), mouse anti‐Connexin 26 (1:200, 13–8100, Invitrogen), rabbit anti‐TMEM16A (1:200, ab53212, Abcam), rabbit anti‐Cav3.2 (1:200, C1868, Sigma‐Aldrich), rabbit anti‐Bestrophin (1:200, ARG10776, arigo), rabbit anti‐Bestrophin 3 (1:200, ARG10775, arigo), Cleaved Caspase‐3 (Asp175) Antibody (1:200, 9661, Cst) , αII‐spectrin (1;200, 803201, BioLegend), rabbit anti‐beta Tubulin (1:200, ab6046, Abcam),EdU (10 µm, ST067,Beyotime) to respectively indicate galectin1 (Lgals1), Calb1 (Calbindin), Connexin 30, and Connexin 26, Ano1 (TMEM16A), Cav3.2, Best1 (Bestrophin), Best3 (Bestrophin 3), Caspase‐3, spectrin, Tubulin, and proliferation in Ko. Secondary antibodies used include Alexa Fluor 488 (absorption: 495 nm, emission: 519 nm) goat anti‐rabbit IgG (H+L) secondary antibody (1:500, A21422, Life) and Alexa Fluor 555 (absorption: 555 nm, emission: 565 nm) goat anti‐mouse IgG (H+L) secondary antibody (1:500, A11008, Life).

The immunostaining of Ko followed the protocol below. After washing with 0.01 M PBS, tissues were then permeabilized with 1% Triton X‐100 for 1 h, and subsequently immersed in blocking solution containing 5% goat serum for 1 h at room temperature. The tissues were incubated with primary antibodies overnight at 4°C, washed with PBS, and incubated with secondary antibodies for 2 h at room temperature. DAPI (F6057; Sigma, St. Louis, MO, USA) was used to label the nucleus. Phalloidin conjugated with fluorescent dyes (ab176756, 1:2,000 diluted; Abcam) was used to label F‐actin in cryosections. After the staining procedures, all samples were observed under a confocal microscope (Zeiss LSM710).

### 4.11 FISH Test

Gene‐specific probes and the ViewRNA ISH Cell Assay Kit (QVC0001) were ordered from Thermo Fisher Scientific.

### 4.12 Western Blotting

Cochleae were harvested, rinsed in cold phosphate‐buffered saline (PBS), and homogenized in lysis buffer (Invitrogen, SD‐001/SN‐002) supplemented with protease and phosphatase inhibitors (1:100 dilution; Epizyme Biomedical Technology Co., Ltd.). Each biological replicate consisted of four cochleae isolated from two mice, with three independent replicates performed per experimental group. The homogenates were centrifuged at 12,000 rpm for 30 min at 4°C, and the resulting supernatants were collected. Protein concentrations were determined using the Omni‐Easy Ready‐to‐Use BCA Protein Assay Kit (ZJ102; EpiZyme) according to the manufacturer's instructions. Equal amounts of protein were loaded onto 6%–15% SDS‐polyacrylamide gels (PG112, PG113; EpiZyme) and transferred onto BioTrace NT nitrocellulose membranes (66485; Pall Corporation, Port Washington, NY, USA). Membranes were blocked with 5% skim milk in 1× TBST buffer (C520009‐0001; Sangon Biotech) for 1 h at room temperature. Following blocking, membranes were incubated overnight at 4°C with primary antibodies. On the following day, after three washes with 1× TBST, membranes were incubated with horseradish peroxidase (HRP)‐conjugated secondary antibodies for 1 h at room temperature. Immunoreactive bands were detected using the Omni‐ECL Femto Light Chemiluminescence Kit (SQ201; EpiZyme), and images were captured using a ChemiDoc XRS imaging system (Bio‐Rad, Richmond, CA, USA). Densitometric analysis was conducted using ImageJ software (National Institutes of Health, Bethesda, MD, USA).

### 4.13 Immunohistochemistry (IHC) Staining

The immunohistochemistry (IHC) staining procedure commences with deparaffinization and rehydration of tissue sections through a graded series of xylene and ethanol solutions. Endogenous peroxidase activity may subsequently be blocked using 3% hydrogen peroxide, if required. Following phosphate‐buffered saline (PBS) rinses, antigen retrieval is performed to expose target epitopes, after which non‐specific binding sites are blocked with normal goat serum. Sections are then incubated with a primary antibody, either at room temperature or overnight at 4°C, depending on the antibody specifications. After washing, an HRP‐conjugated secondary antibody is applied and incubated at 37°C. Immunoreactivity is visualized using 3,3'‐diaminobenzidine (DAB) chromogen, with reaction progress monitored microscopically. The staining process concludes with distilled water rinsing, counterstaining with hematoxylin, differentiation, dehydration through a graded alcohol series, clearing in xylene, and coverslip mounting.

### 4.14 ABR Test

The body temperature was maintained at 37°C using a thermostatically controlled heating pad following anesthesia induction in mice with 1% sodium pentobarbital (75 mg kg^−1^). Auditory brainstem responses (ABRs) were recorded using hardware and software from Tucker‐Davis Technologies (TDT System III; Alachua, FL, USA). Acoustic stimuli were delivered via an MF1a broadband speaker at a stimulation rate of 21.1 stimuli per second, presented to one ear through a plastic tubing in a closed‐field configuration. Subdermal electrodes were positioned at the cranial vertex (active electrode), beneath the mastoid of the tested ear (reference electrode), and in the contralateral hindlimb (ground electrode). Stimulus intensity was initially set at 90 dB sound pressure level (SPL) and decreased in 5 dB decrements down to 0 dB SPL. The ABR threshold was defined as the lowest intensity at which wave III could no longer be reliably identified across test frequencies ranging from 4 to 45.2 kHz.

### 4.15 Data Analysis

The Ca^2+^ wave was identified by two pre‐defined thresholds: amplitude over 1.2 folds of the fluorescence baseline and propagation area > 10 µm^2^ to eliminate possible false‐positive Ca^2+^ waves caused by the fluorescence fluctuation during microscopy. The average fluorescence intensity and propagation area of Ca^2+^ waves, both changing over time were calculated using ImageJ software one by one. The fluorescence baseline was the average of the fluorescence intensity obtained from multiple images (about 10 frames) in the absence of a Ca^2+^ wave. The frequency, amplitude, and FWHM of each event were acquired by peak analysis using Origin software. The velocity was defined as the average increasing area of Ca^2+^ wave per second. For the calculation of the Ca^2+^ events ratio, in Figure 1J, it refers to the proportion of spontaneous Ca^2+^ waves (non‐hotspot) occurring within 30 s after Ca^2+^ lightning flashes, relative to the total such waves. In Figure 1K, it refers to the temporal distribution of spontaneous waves (non‐hotspot) across defined intervals as a percentage of total spontaneous waves. In Figure 1L, it refers to the proportion of spontaneous Ca^2+^ waves (non‐hotspot) with different delays relative to hotspot the Ca^2+^ wave to all spontaneous waves. For the normalization processes in Figure 4F,I,L, each cochlea was randomly stimulated three times by a femtosecond laser before the treatment. Then the average amplitude or area of Ca^2+^ waves calculated from the three trials was used as the normalized standard. We used box whiskers (min to max) to show the data distribution and analyzed it with GraphPad Prism 7 software through the two‐tailed t‐test except where stated otherwise. The confidence interval for t‐test was set as 95%. A Mann–Whitney U test was used for the analysis of data that did not follow a normal distribution. A chi‐squared test (χ^2^) was used for the comparison of rates ≥ 2.

## Author Contributions

H.H., H.‐B.S., and S.‐K.Y. conceived the study and supervised the work. Q.M. prepared the animals, performed surgeries and molecular biology studies. H.‐P.W. set up the optical system, performed the cochlear microscopy, and analyzed the data. K.L., M.‐X.L. and L.‐N.G performed electrophysiological studies. H.‐P.W. and Q.M., and H.H. prepared the figures. All authors discussed and analyzed the data. H.H. and H.‐B.S. prepared the draft. L.‐Y.W., H.‐B.S., S.‐K.Y., K.‐M.S., and H.H. revised the manuscript.

## Ethics Statement

All experimental procedures regarding the care and use of animals were reviewed and approved by the Animal Ethics Committee of Shanghai Sixth People's Hospital (Approval Number: 2019‐0246). All efforts were made to minimize possible pain and discomfort of the animals during the experimental procedures.

(Ethical approval statements, assigned numbers, and patient consent statements are required only for research articles involving animal experiments or human subjects/clinical trials)

(Trial registration numbers are required only for research articles involving clinical trials)

## Conflicts of Interest

The authors declare no conflicts of interests.

## Supporting information




**Supporting File 1**: exp270205‐sup‐0001‐SuppMat.docx.

**Supporting File 2**: exp270205‐sup‐0002‐MovieS1.mp4.

**Supporting File 3**: exp270205‐sup‐0003‐MovieS2.mp4.

**Supporting File 4**: exp270205‐sup‐0004‐MovieS3.mp4.

**Supporting File 5**: exp270205‐sup‐0005‐MovieS4.mp4.

## Data Availability

The data that support the findings of this study are available from the corresponding author upon reasonable request.

## References

[1] A. G. Blankenship and M. B. Feller , “Mechanisms Underlying Spontaneous Patterned Activity in Developing Neural Circuits,” Nature Reviews Neuroscience 11 (2010): 18–29, 10.1038/nrn2759. 19953103 PMC2902252

[2] A. D. Huberman , M. B. Feller , and B. Chapman , “Mechanisms Underlying Development of Visual Maps and Receptive Fields,” Annual Review of Neuroscience 31 (2008): 479–509, 10.1146/annurev.neuro.31.060407.125533. PMC2655105 18558864

[3] S. Sun , T. Babola , G. Pregernig , et al., “Hair Cell Mechanotransduction Regulates Spontaneous Activity and Spiral Ganglion Subtype Specification in the Auditory System,” Cell 174 (2018): 1247–1263.e15, 10.1016/j.cell.2018.07.008. 30078710 PMC6429032

[4] N. Antón‐Bolaños , A. Sempere‐Ferràndez , T. Guillamón‐Vivancos , et al., “Prenatal Activity From Thalamic Neurons Governs the Emergence of Functional Cortical Maps in Mice,” Science 364 (2019): 987–990. 31048552 10.1126/science.aav7617 PMC7611000

[5] T. A. Babola , S. Li , A. Gribizis , et al., “Homeostatic Control of Spontaneous Activity in the Developing Auditory System,” Neuron 99 (2018): 511–524.e5, 10.1016/j.neuron.2018.07.004. 30077356 PMC6100752

[6] B. R. Shrestha , C. Chia , L. Wu , S. G. Kujawa , M. C. Liberman , and L. V. Goodrich , “Sensory Neuron Diversity in the Inner Ear Is Shaped by Activity,” Cell 174 (2018): 1229–1246.e17, 10.1016/j.cell.2018.07.007. 30078709 PMC6150604

[7] N. X. Tritsch , E. Yi , J. E. Gale , E. Glowatzki , and D. E. Bergles , “The Origin of Spontaneous Activity in the Developing Auditory System,” Nature 450 (2007): 50–55, 10.1038/nature06233. 17972875

[8] N. X. Tritsch , A. Rodríguez‐Contreras , T. T. Crins , H. C. Wang , J. G. Borst , and D. E. Bergles , “Calcium Action Potentials in Hair Cells Pattern Auditory Neuron Activity Before Hearing Onset,” Nature Neuroscience 13 (2010): 1050–1052, 10.1038/nn.2604. 20676105 PMC2928883

[9] H. C. Wang , C. C. Lin , R. Chong , et al., “Spontaneous Activity of Cochlear Hair Cells Triggered by Fluid Secretion Mechanism in Adjacent Support Cells,” Cell 163 (2015): 1348–1359, 10.1016/j.cell.2015.10.070. 26627734 PMC4671825

[10] T. A. Babola , C. J. Kersbergen , H. C. Wang , and D. E. Bergles , “Purinergic Signaling in Cochlear Supporting Cells Reduces Hair Cell Excitability by Increasing the Extracellular Space,” Elife 9 (2020): e52160, 10.7554/eLife.52160. 31913121 PMC7015667

[11] N. X. Tritsch and D. E. Bergles , “Developmental Regulation of Spontaneous Activity in the Mammalian Cochlea,” The Journal of Neuroscience 30 (2010): 1539–1550, 10.1523/JNEUROSCI.3875-09.2010. 20107081 PMC2814371

[12] G. Sendin , J. Bourien , F. Rassendren , J. L. Puel , and R. Nouvian , “Spatiotemporal Pattern of Action Potential Firing in Developing Inner Hair Cells of the Mouse Cochlea,” Proceedings of the National Academy of Sciences 111 (2014): 1999–2004, 10.1073/pnas.1319615111. PMC3918831 24429348

[13] M. J. Moglie , P. A. Fuchs , A. B. Elgoyhen , and J. D. Goutman , “Compartmentalization of Antagonistic Ca^2+^ Signals in Developing Cochlear Hair Cells,” Proceedings of the National Academy of Sciences 115 (2018): E2095, 10.1073/pnas.1719077115. PMC5834711 29439202

[14] D. Oliver , N. Klöcker , J. Schuck , T. Baukrowitz , J. P. Ruppersberg , and B. Fakler , “Gating of Ca^2+^‐Activated K^+^ Channels Controls Fast Inhibitory Synaptic Transmission at Auditory Outer Hair Cells,” Neuron 26 (2000): 595–601, 10.1016/S0896-6273(00)81197-6. 10896156

[15] K. Castaño‐González , C. Köppl , and S. J. Pyott , “The Crucial Role of Diverse Animal Models to Investigate Cochlear Aging and Hearing Loss,” Hearing Research 445 (2024): 108989, 10.1016/j.heares.2024.108989. 38518394

[16] C. A. Vivaldo , J. Lee , M. Shorkey , A. Keerthy , and G. Rothschild , “Auditory Cortex Ensembles Jointly Encode Sound and Locomotion Speed to Support Sound Perception During Movement,” PLoS Biology 21 (2023): e3002277, 10.1371/journal.pbio.3002277. 37651461 PMC10499203

[17] R. Hertzano , A. A. Dror , M. Montcouquiol , et al., “Lhx3, a LIM Domain Transcription Factor, Is Regulated by Pou4f3 in the Auditory but Not in the Vestibular System,” European Journal of Neuroscience 25 (2007): 999–1005, 10.1111/j.1460-9568.2007.05332.x. 17331196

[18] O. Michel , A. Hess , W. Bloch , A. Schmidt , E. Stennert , and K. Addicks , “Immunohistochemical Detection of Vascular Endothelial Growth Factor (VEGF) and VEGF Receptors Flt‐1 and KDR/Flk‐1 in the Cochlea of guinea Pigs,” Hearing Research 155 (2001): 175–180, 10.1016/S0378-5955(01)00262-3. 11335087

[19] T. M. Coate , S. Raft , X. Zhao , A. K. Ryan , E. B. Crenshaw 3rd , and M. W. Kelley , “Otic Mesenchyme Cells Regulate Spiral Ganglion Axon Fasciculation Through a Pou3f4/EphA4 Signaling Pathway,” Neuron 73 (2012): 49–63, 10.1016/j.neuron.2011.10.029. 22243746 PMC3259535

[20] B. H. Hartman , R. Durruthy‐Durruthy , R. D. Laske , S. Losorelli , and S. Heller , “Identification and Characterization of Mouse Otic Sensory Lineage Genes,” Frontiers in Cellular Neuroscience 9 (2015): 79, 10.3389/fncel.2015.00079. 25852475 PMC4365716

[21] D. Forrest , L. C. Erway , L. Ng , R. Altschuler , and T. Curran , “Thyroid Hormone Receptor β Is Essential for Development of Auditory Function,” Nature Genetics 13 (1996): 354–357, 10.1038/ng0796-354. 8673137

[22] A. Rusch , L. Ng , R. Goodyear , et al., “Retardation of Cochlear Maturation and Impaired Hair Cell Function Caused by Deletion of all Known Thyroid Hormone Receptors,” The Journal of Neuroscience 21 (2001): 9792–9800, 10.1523/JNEUROSCI.21-24-09792.2001. 11739587 PMC6763054

[23] P. Cheng , X. Tian , W. Tang , et al., “Direct Control of Store‐Operated Calcium Channels by Ultrafast Laser,” Cell Research 31 (2021): 758–772, 10.1038/s41422-020-00463-9. 33469157 PMC8249419

[24] P. Liu , J. Gong , X. Ding , et al., “The L‐type Ca(2+) Channel Blocker Nifedipine Inhibits Mycelial Growth, Sporulation, and Virulence of Phytophthora Capsici,” Frontiers in Microbiology 7 (2016): 1236. 27540377 10.3389/fmicb.2016.01236 PMC4972815

[25] C. Seto , K. Toyoda , K. Inada , K. Oka , and E. Ito , “Influence of Gap Junctions Upon Ca^2+^ Propagation From Stimulated Keratinocytes to DRG Neurons,” Biophysics and Physicobiology 19 (2022): e190041, 10.2142/biophysico.bppb-v19.0041. 36349331 PMC9592570

[26] S. Y. Hong , Y. Y. Yang , S. G. Wang , and B. L. Qin , “Inhibition of AT1R/IP3/IP3R‐Mediated Ca^2+^ Release Protects Against Calcium Oxalate Crystals‐Induced Renal Oxidative Stress,” Chemico‐Biological Interactions 382 (2023): 110636, 10.1016/j.cbi.2023.110636. 37454925

[27] T. Jiao , A. Collado , A. Mahdi , et al., “Erythrocytes From Patients With ST‐Elevation Myocardial Infarction Induce Cardioprotection Through the Purinergic P2Y13 Receptor and Nitric Oxide Signaling,” Basic Research in Cardiology 117 (2022): 46, 10.1007/s00395-022-00953-4. 36112326 PMC9481504

[28] V. S. Kuzmin , K. B. Pustovit , and D. V. Abramochkin , “Effects of Exogenous Nicotinamide Adenine Dinucleotide (NAD+) in the Rat Heart Are Mediated by P2 Purine Receptors,” Journal of Biomedical Science 23 (2016): 50, 10.1186/s12929-016-0267-y. 27350532 PMC4924331

[29] M. Sedeeq , A. Maklad , T. Dutta , et al., “T‐Type Calcium Channel Inhibitors Induce Apoptosis in Medulloblastoma Cells Associated With Altered Metabolic Activity,” Molecular Neurobiology 59 (2022): 2932–2945, 10.1007/s12035-022-02771-0. 35243582 PMC9016057

[30] A. Visa , L. Alza , C. Cantí , and J. Herreros , “Tetralol Derivative NNC‐55‐0396 Induces Glioblastoma Cell Death by Activating IRE1α, JNK1 and Calcium Signaling,” Biomedicine & Pharmacotherapy 149 (2022): 112881, 10.1016/j.biopha.2022.112881. 35367758

[31] C. O. Malécot , “Low Voltage‐Activated Channels in Rat Pulmonary Vein Cardiomyocytes: Coexistence of a Non‐Selective Cationic Channel and of T‐type Ca Channels,” Pflugers Archiv: European Journal of Physiology 472 (2020): 1019. 32556635 10.1007/s00424-020-02413-1

[32] T. Ohkubo and J. Yamazaki , “T‐type Voltage‐Activated Calcium Channel Cav3.1, but Not Cav3.2, Is Involved in the Inhibition of Proliferation and Apoptosis in MCF‐7 human Breast Cancer Cells,” International Journal of Oncology 41 (2012): 267–275. 22469755 10.3892/ijo.2012.1422

[33] J. Jansen , M. Qiao , L. Hertz , et al., “Mechanistic Ion Channel Interactions in Red Cells of Patients With Gárdos Channelopathy,” Blood Advances 5 (2021): 3303–3308, 10.1182/bloodadvances.2020003823. 34468723 PMC8525243

[34] V. Yarotskyy and K. S. Elmslie , “Roscovitine Inhibits CaV3.1 (T‐Type) Channels by Preferentially Affecting Closed‐State Inactivation,” The Journal of Pharmacology and Experimental Therapeutics 340 (2012): 463–472, 10.1124/jpet.111.187104. 22088954 PMC3263959

[35] B. Mlinar and J. J. Enyeart , “Block of Current Through T‐Type Calcium Channels by Trivalent Metal Cations and Nickel in Neural Rat and Human Cells,” The Journal of Physiology 469 (1993): 639–652, 10.1113/jphysiol.1993.sp019835. 8271221 PMC1143892

[36] Y. Li , T. Yuan , B. Huang , et al., “Structure of Human NaV1.6 Channel Reveals Na^+^ Selectivity and Pore Blockade by 4,9‐Anhydro‐Tetrodotoxin,” Nature Communications 14 (2023): 1030, 10.1038/s41467-023-36766-9. PMC9950489 36823201

[37] J. Heijman , X. Zhou , S. Morotti , et al., “Enhanced Ca^2+^‐Dependent SK‐Channel Gating and Membrane Trafficking in Human Atrial Fibrillation,” Circulation Research 132 (2023): e116, 10.1161/CIRCRESAHA.122.321858. 36927079 PMC10147588

[38] X. Huang , X. Jin , G. Huang , et al., “Structural Basis for High‐Voltage Activation and Subtype‐Specific Inhibition of Human Na v 1.8,” Proceedings of the National Academy of Sciences 119 (2022): e2208211119, 10.1073/pnas.2208211119. PMC9335304 35858452

[39] Y. Liu , H. Zhang , D. Huang , et al., “Characterization of the Effects of Cl− Channel Modulators on TMEM16A and Bestrophin‐1 Ca^2+^ Activated Cl− Channels,” Pflügers Archiv—European Journal of Physiology 467 (2015): 1417–1430, 10.1007/s00424-014-1572-5. 25078708

[40] J. Danielsson , J. Perez‐Zoghbi , K. Bernstein , et al., “Antagonists of the TMEM16A Calcium‐Activated Chloride Channel Modulate Airway Smooth Muscle Tone and Intracellular Calcium,” Anesthesiology 123 (2015): 569–581, 10.1097/ALN.0000000000000769. 26181339 PMC4543527

[41] K. Tan‐No , A. Taira , K. Wako , et al., “Intrathecally Administered Spermine Produces the Scratching, Biting and Licking Behaviour in Mice,” Pain 86 (2000): 55–61, 10.1016/S0304-3959(99)00312-7. 10779660

[42] J. E. Libbey , T. J. Hanak , D. J. Doty , K. S. Wilcox , and R. S. Fujinami , “NBQX, a Highly Selective Competitive Antagonist of AMPA and KA Ionotropic Glutamate Receptors, Increases Seizures and Mortality Following Picornavirus Infection,” Experimental Neurology 280 (2016): 89–96, 10.1016/j.expneurol.2016.04.010. 27072529 PMC4860063

[43] A. Quesada , P. H. Bui , G. E. Homanics , O. Hankinson , and A. Handforth , “Comparison of mibefradil and Derivative NNC 55‐0396 Effects on Behavior, Cytochrome P450 Activity, and Tremor in Mouse Models of Essential Tremor,” European Journal of Pharmacology 659 (2011): 30–36, 10.1016/j.ejphar.2011.01.004. 21256842 PMC3988263

[44] Y. Yabuki , K. Matsuo , H. Izumi , et al., “Pharmacological Properties of SAK3, a Novel T‐type Voltage‐Gated Ca^2+^ Channel Enhancer,” Neuropharmacology 117 (2017): 1–13, 10.1016/j.neuropharm.2017.01.011. 28093211

[45] V. Munnamalai and D. M. Fekete , “Notch‐Wnt‐Bmp Crosstalk Regulates Radial Patterning in the Mouse Cochlea in a Spatiotemporal Manner,” Development 143 (2016): 4003–4015, 10.1242/dev.139469. 27633988 PMC5117145

[46] C. J. Kersbergen , T. A. Babola , J. Rock , and D. E. Bergles , “Developmental Spontaneous Activity Promotes Formation of Sensory Domains, Frequency Tuning and Proper Gain in Central Auditory Circuits,” Cell Reports 41 (2022): 111649, 10.1016/j.celrep.2022.111649. 36384119 PMC9730452

[47] A. Maul , A. K. Huebner , N. Strenzke , et al., “The Cl–Channel TMEM16A Is Involved in the Generation of Cochlear Ca^2+^ Waves and Promotes the Refinement of Auditory Brainstem Networks in Mice,” Elife 11 (2022): e72233, 10.7554/eLife.72251. PMC8871368 35129434

[48] J. Chen , D. Gao , L. Sun , and J. Yang , “Kölliker's Organ‐Supporting Cells and Cochlear Auditory Development,” Frontiers in Molecular Neuroscience 15 (2022): 1031989, 10.3389/fnmol.2022.1031989. 36304996 PMC9592740

[49] V. Borse , T. Kaur , A. Hinton , K. Ohlemiller , and M. E. Warchol , “Programmed Cell Death Recruits Macrophages into the Developing Mouse Cochlea,” Cell and Developmental Biology 9 (2021): 777836. 10.3389/fcell.2021.777836 PMC8696258 34957108

[50] L. Sun , D. Gao , J. Chen , et al., “Failure of Hearing Acquisition in Mice With Reduced Expression of Connexin 26 Correlates With the Abnormal Phasing of Apoptosis Relative to Autophagy and Defective ATP‐Dependent Ca^2+^ Signaling in Kölliker's Organ,” Frontiers in Cellular Neuroscience 16 (2022): 816079, 10.3389/fncel.2022.816079. 35308122 PMC8928193

[51] C. J. Kersbergen and D. E. Bergles , “Priming central Sound Processing Circuits Through Induction of Spontaneous Activity in the Cochlea Before Hearing Onset,” Trends in Neurosciences 47 (2024): 522–537, 10.1016/j.tins.2024.04.007. 38782701 PMC11236524

[52] H. P. Xu , T. J. Burbridge , M. Ye , et al., “Retinal Wave Patterns Are Governed by Mutual Excitation Among Starburst Amacrine Cells and Drive the Refinement and Maintenance of Visual Circuits,” The Journal of Neuroscience 36 (2016): 3871–3886, 10.1523/JNEUROSCI.3549-15.2016. 27030771 PMC4812142

[53] D. P. Kelsell , J. Dunlop , H. P. Stevens , et al., “Connexin 26 Mutations in Hereditary Non‐Syndromic Sensorineural Deafness,” Nature 387 (1997): 80–83, 10.1038/387080a0. 9139825

[54] H. C. Hartzell , Z. Qu , K. Yu , Q. Xiao , and L. T. Chien , “Molecular Physiology of Bestrophins: Multifunctional Membrane Proteins Linked to Best Disease and Other Retinopathies,” Physiological Reviews 88 (2008): 639–672, 10.1152/physrev.00022.2007. 18391176

[55] L. Kolla , M. C. Kelly , Z. F. Mann , et al., “Characterization of the Development of the Mouse Cochlear Epithelium at the Single Cell Level,” Nature Communications 11 (2020): 2389, 10.1038/s41467-020-16113-y. PMC7221106 32404924

[56] N. Xie , A. Landin Malt , A. Adylkhan , et al., “Wnt7b acts in Concert With Wnt5a to Regulate Tissue Elongation and Planar Cell Polarity via Noncanonical Wnt Signaling,” Proceedings of the National Academy of Sciences 121 (2024): e2405217121, 10.1073/pnas.2405217121. PMC11363310 39172791

[57] J. F. Mulvaney , E. M. Layman , F. Feroze‐Merzoug , et al., “Wnt/PKC Signaling Inhibits Sensory Hair Cell Formation in the Developing Mammalian Cochlea,” Cells 14 (2025): 888, 10.3390/cells14120888. 40558515 PMC12191204

[58] A. Lundt , R. Seidel , J. Soós , et al., “Cav3.2 T‐Type Calcium Channels Are Physiologically Mandatory for the Auditory System,” Neuroscience 409 (2019): 81–100, 10.1016/j.neuroscience.2019.04.024. 31029730

[59] D. Ding , J. He , B. L. Allman , et al., “Cisplatin Ototoxicity in Rat Cochlear Organotypic Cultures,” Hearing Research 282 (2011): 196–203, 10.1016/j.heares.2011.08.002. 21854840 PMC3230738

[60] A. Butler , P. Hoffman , P. Smibert , E. Papalexi , and R. Satija , “Integrating Single‐Cell Transcriptomic Data Across Different Conditions, Technologies, and Species,” Nature Biotechnology 36 (2018): 411–420, 10.1038/nbt.4096. PMC6700744 29608179

[61] X. Qiu , Q. Mao , Y. Tang , et al., “Reversed Graph Embedding Resolves Complex Single‐Cell Trajectories,” Nature Methods 14 (2017): 979–982, 10.1038/nmeth.4402. 28825705 PMC5764547

[62] K. Street , D. Risso , R. B. Fletcher , et al., “Slingshot: Cell Lineage and Pseudotime Inference for Single‐Cell Transcriptomics,” BMC Genomics 19 (2018): 477, 10.1186/s12864-018-4772-0. 29914354 PMC6007078

[63] K. Van den Berge , H. Roux de Bézieux , K. Street , et al., “Trajectory‐Based Differential Expression Analysis for Single‐Cell Sequencing Data,” Nature Communications 11 (2020): 1201. 10.1038/s41467-020-14766-3 PMC7058077 32139671

