# Supplementary material for: Triggered Calcium Lightning Programs Cochlear Development
Source: Exploration (Beijing). 2026 Jul 26:70205. Online ahead of print. doi: 10.1002/exp2.70205 (PMC13402436; doi:10.1002/exp2.70205)
Supplement: Supplementary file 1 — Supporting File 1: exp270205‐sup‐0001‐SuppMat.docx. [file EXP2-9999-0-s005.docx]

**Triggered calcium lightning programs cochlear development**

**Supplementary Figures**


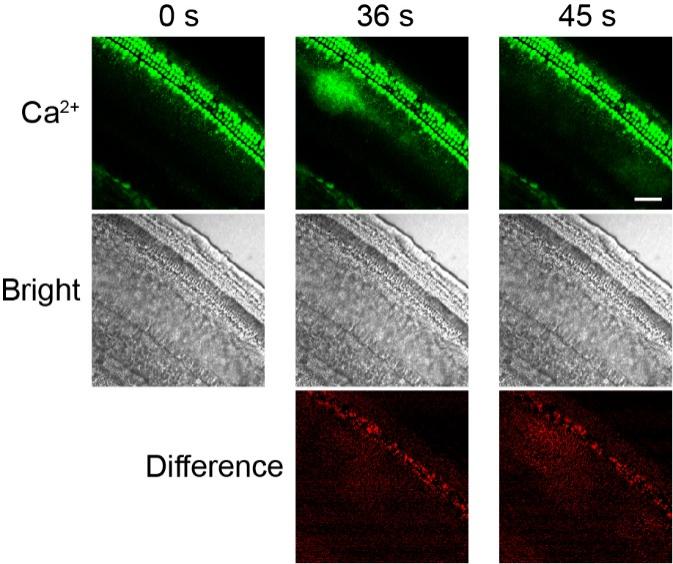


**Supplementary figure 1 Simultaneous fluorescent and bright-field microscopy of cochlear Ca^2+^ dynamics and tissue.** Bottom: calculated differences between bright-field images at indicated time slots and it at 0 s. Scale bar: 50 μm.


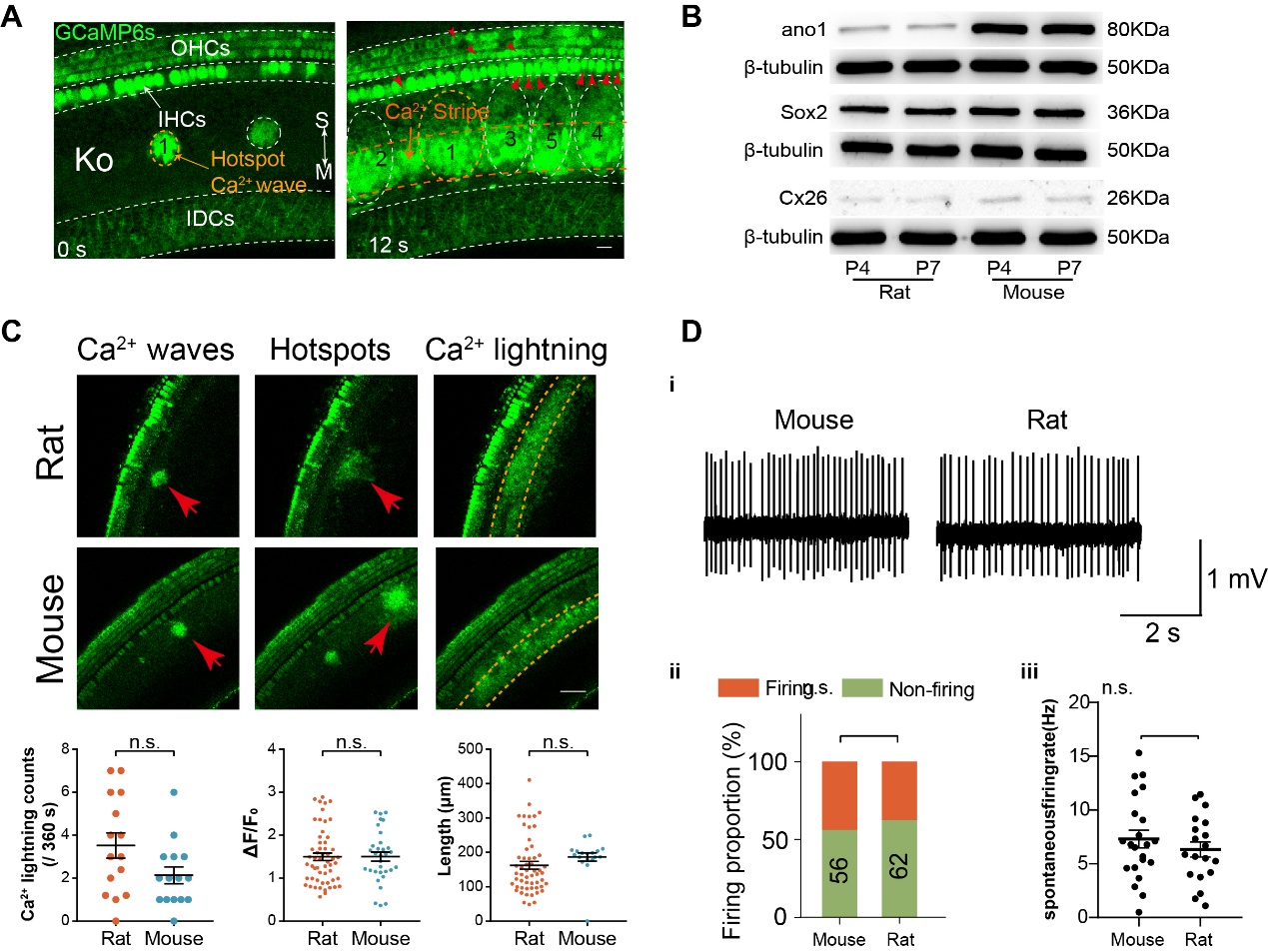


**Supplementary figure2 Verification of Species Consistency in Rats and Mice. A**. consistence of rats and mice in key biomarkers of cochlear early development, including Ano1, connexin 26, and SOX2 at P4 and P7 respectively. **B**. Comparison of Ca^2+^ imaging in the cochleae of rats and mice, including Ca^2+^ waves, hotspots, and Ca^2+^ lightning. Right: Comparison of the frequency, ΔF/F_0_ and length of Ca^2+^ lightning between rats and mice. Scale bar: 50 μm. **C**. Cochlear nucleus (CN) neurons in mice and rats exhibited similar spontaneous firings (SFs) in the early postnatal stage (P4–P7). i, Representative raw traces showing cell-attached recordings of SFs in current-clamp mode. ii, The proportion of spontaneous firing neurons in the cochlear nuclei from C57 mice and SD rats. iii: The frequency of spontaneous firing in the firing neurons in cochlear nuclei from C57 mice and SD rats. [C57BL/6J (n = 22 cells from 3 mice) 7.301 ± 3.892 Hz vs Sprague-Dawley (n = 19 cells from 3 rats) 6.325 ± 3.086 Hz]. Error bars represent means ± SED, independent-samples t test and chi-squared test (χ2). D. Ca^2+^ lightning and following spontaneous Ca^2+^ waves indicated by GCaMP6s in the cochlea of transgenic CAG-GCaMP6s mice, recorded from 0 to 12 s. The numbers 1-5 indicate the sequence of Ca^2+^ waves. Red arrows: Ca^2+^ transients of IHCs and OHCs.


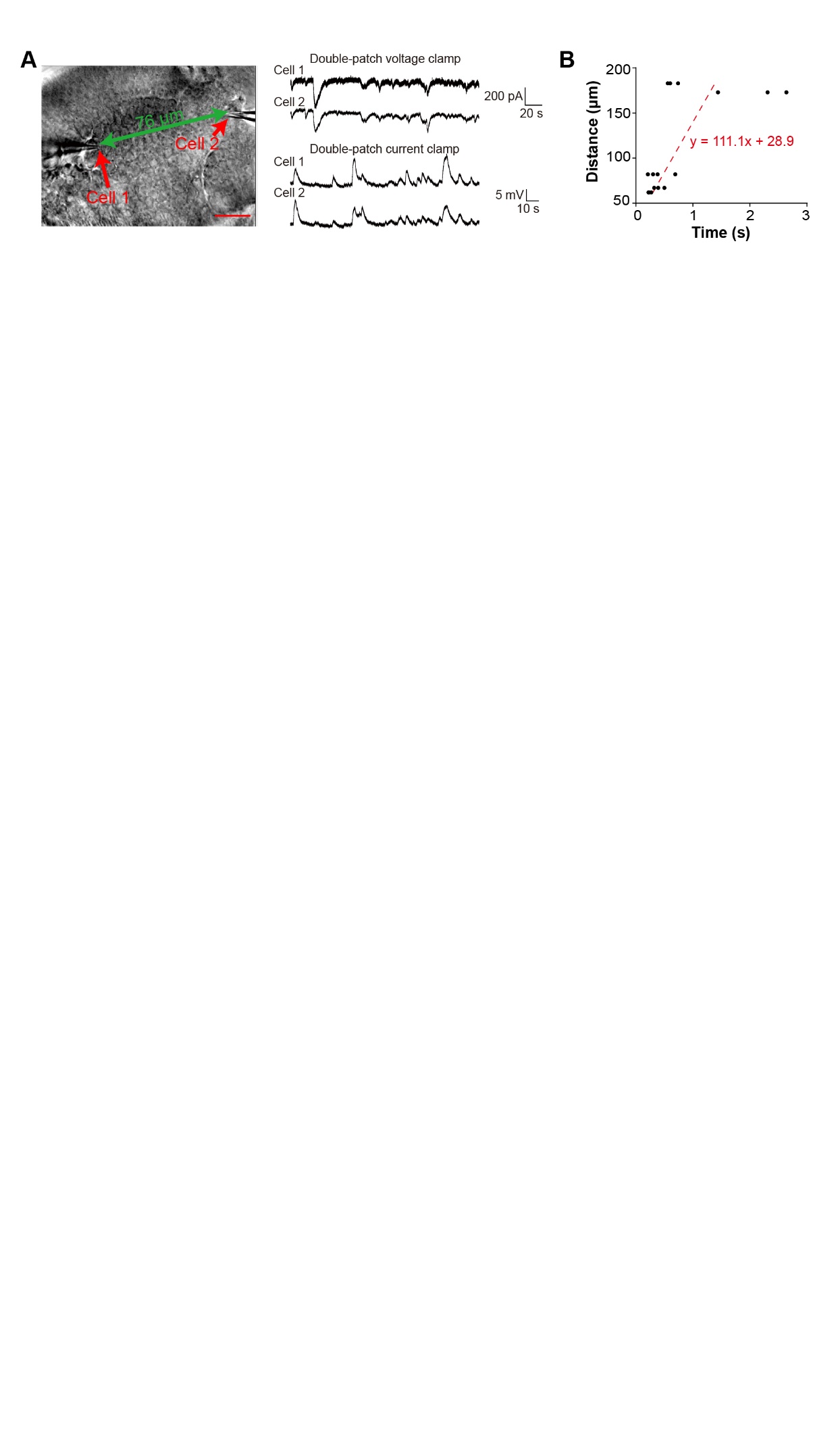


**Supplementary figure 3 Double patch-clamp recordings of two ISCs.** **A.** The simultaneous electrophysiological signals from two ISCs measured by double patch-clamp (*n* = 16 records in 5 independent trials from 5 cochleae of P4 to P7). Bar: 20 μm. **B.** Propagation velocity of the Ca^2+^ measured by double patch clamping. Dashed line: the fitted line. The cochlea used in Supplementary Fig. 3 were all from rats.


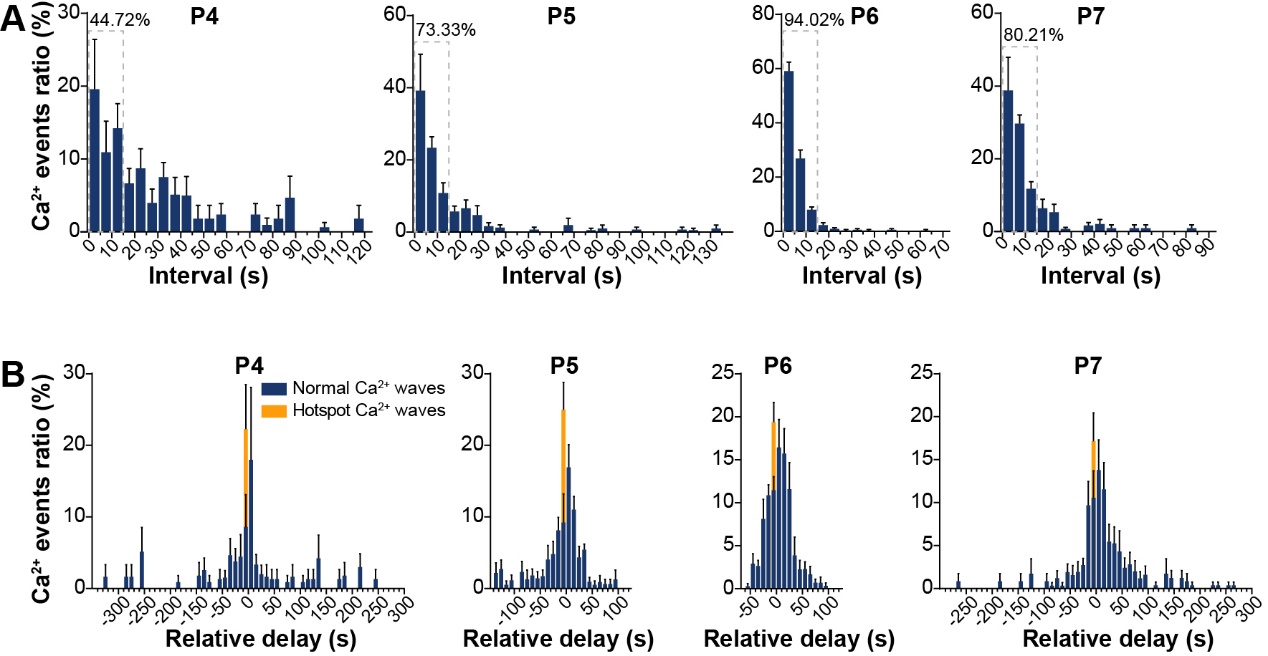


**Supplementary figure 4 The distribution of time intervals between spontaneous Ca^2+^ events at each developmental stage from P4 to P7.** **A.** The distribution of Ca^2+^ events with different intervals between them at different developmental stages (for fig **A**, **B**, *n* = 5, 5, 5, 5 cochleae respectively for P4 to P7). **B.** The distribution of the time delay between Ca^2+^ events and hotspot Ca^2+^ initiation at each developmental stage from P4 to P7. The cochlea used in Supplementary fig. 4 were all from rats.

**
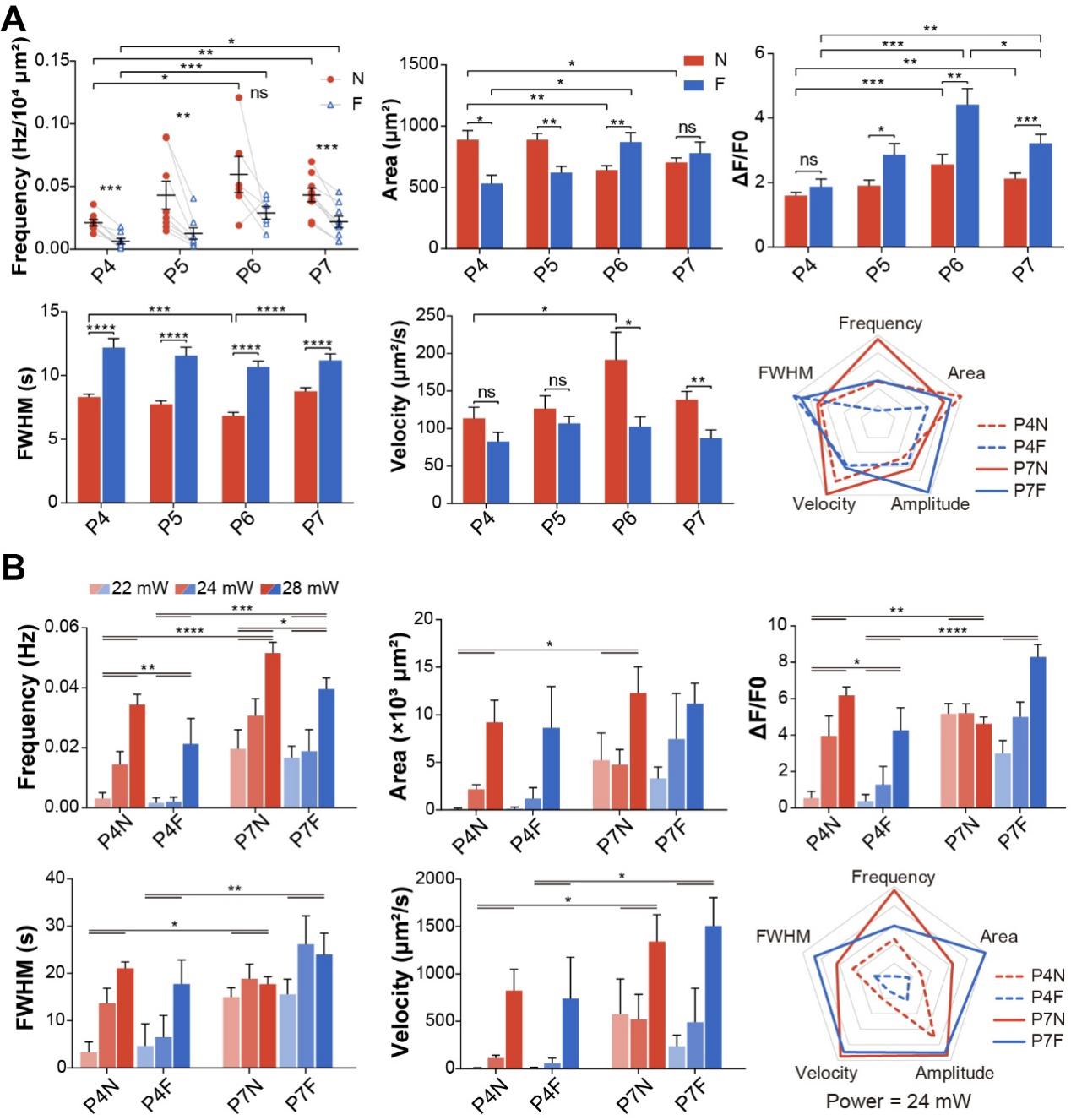
**

**Supplementary figure 5 Characteristics of Ca^2+^ waves in N and F zones.** **A.** Characteristics of spontaneous Ca^2+^ waves in N and F zones from P4 to P7. Right-bottom: the Radar Chart shows the change in the mean parameter of the Ca^2+^ waves from P4 to P7. **B.** The laser-induced Ca^2+^ waves evoked by different laser powers in N and F zones in Ko from P4 to P7. Right-bottom: the Radar Chart shows the change in the mean parameter of the Ca^2+^ waves induced by laser stimulation. (*n* = 7, 4, 4 cochleae respectively per power level for P4 N zone; *n* = 5, 4, 6 cochleae respectively per power level for P4 F zone; *n* = 4, 3, 4 cochleae respectively per power level for P7 N zone; *n* = 4 cochleae for all power levels for P7 F zone). The cochlea used in Supplementary fig. 5 were all from rats.

**
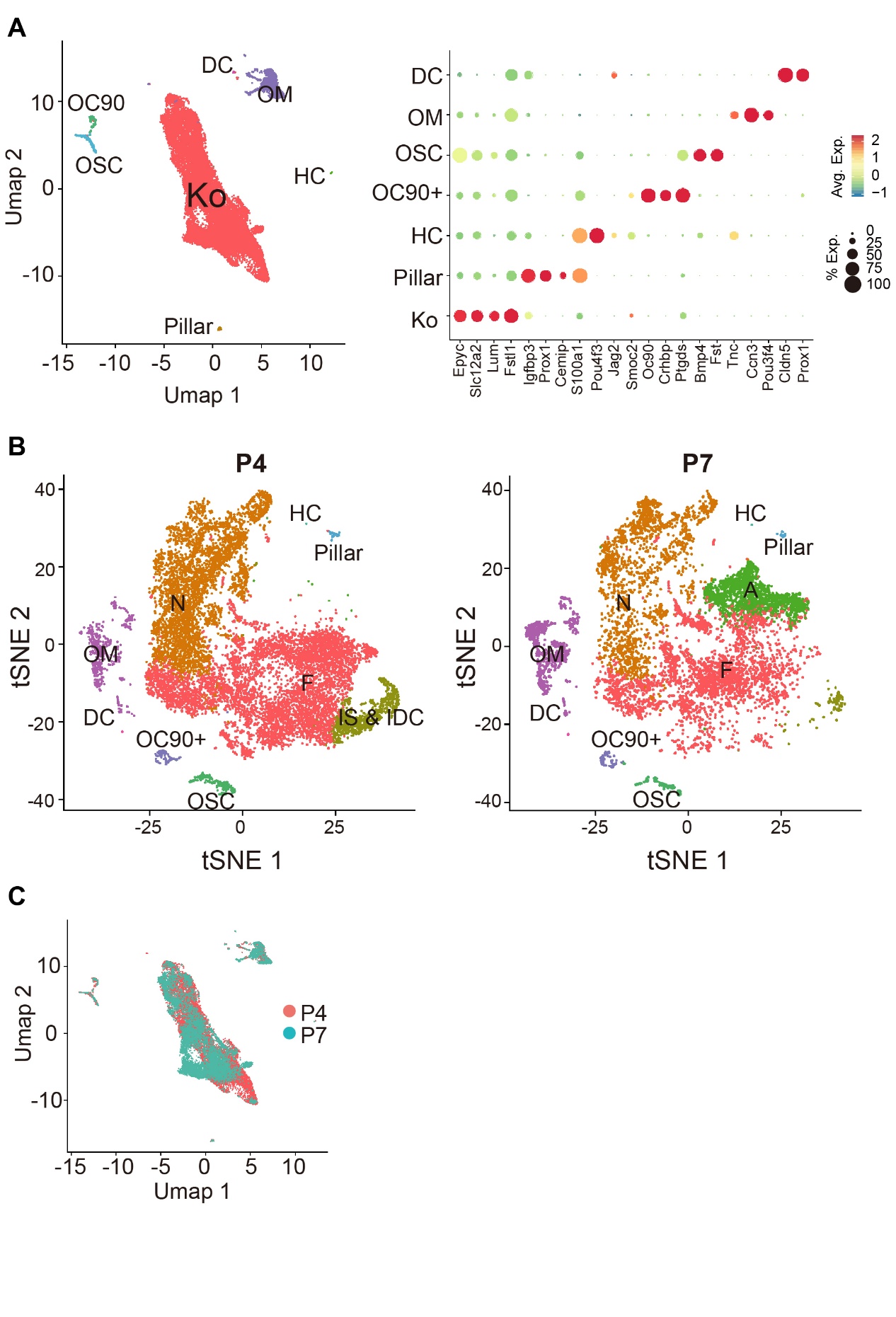
**

**Supplementary figure 6 The cell clustering of all the samples**. **A.** The major part of the sample come solely from Ko. Other cell types in the cochlea could also be identified. Right panel: the featured genes for identification of cell clusters of the total samples. **B.** The cell clusters by tSNE method at P4 and P7 respectively. **C.** The cells come from samples at P4 and P7 respectively. The cochlea used in Supplementary fig. 6 were all from rats.

**
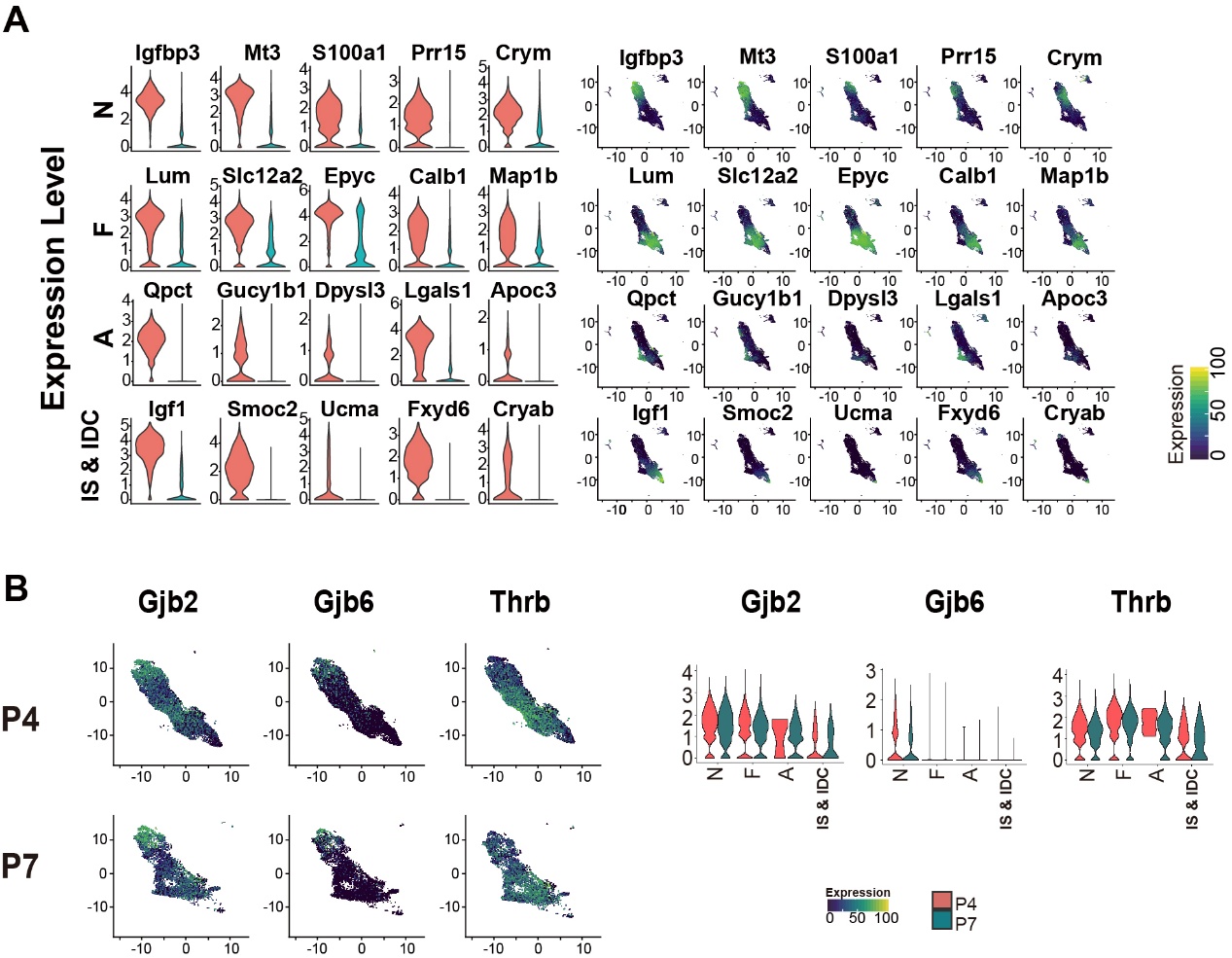
**

**Supplementary figure 7 Expression of each marker in cluster N, F, and A. A.** Violin plots and featured plots of each gene maker indicate expression values for the top five genes for each cell type by comparison with all other cell types. **B.** Expression mapping and Violin plots of selected genes including *Gjb2* in cell clusters at P4 and P7. The cochlea used in Supplementary fig. 7 were all from rats.

**
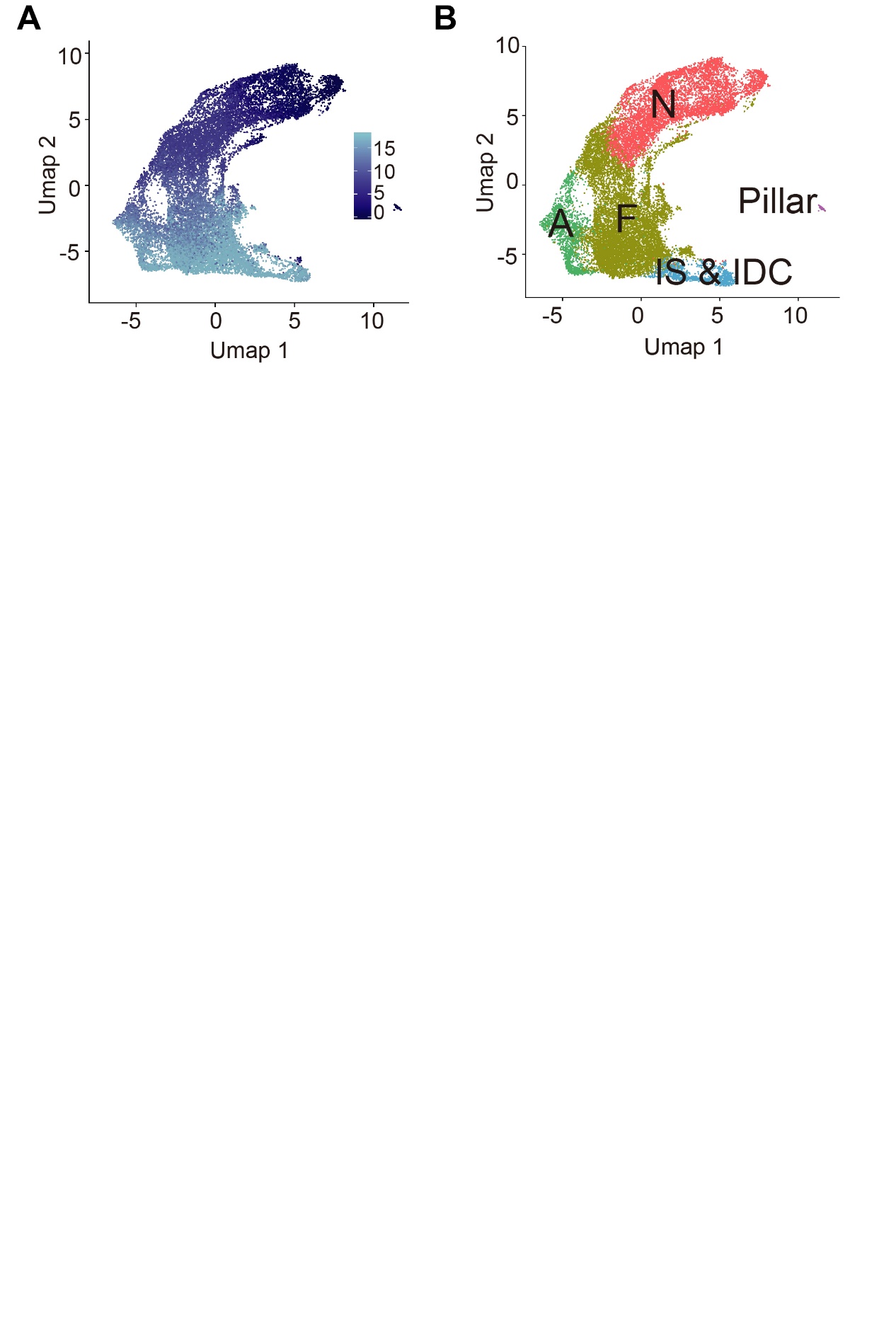
**

**Supplementary figure 8 The trajectory analysis of the cells by clusters (A) and the total sample to show the pseudotime analysis (B).** The cochlea used in Supplementary fig. 8 were all from rats.


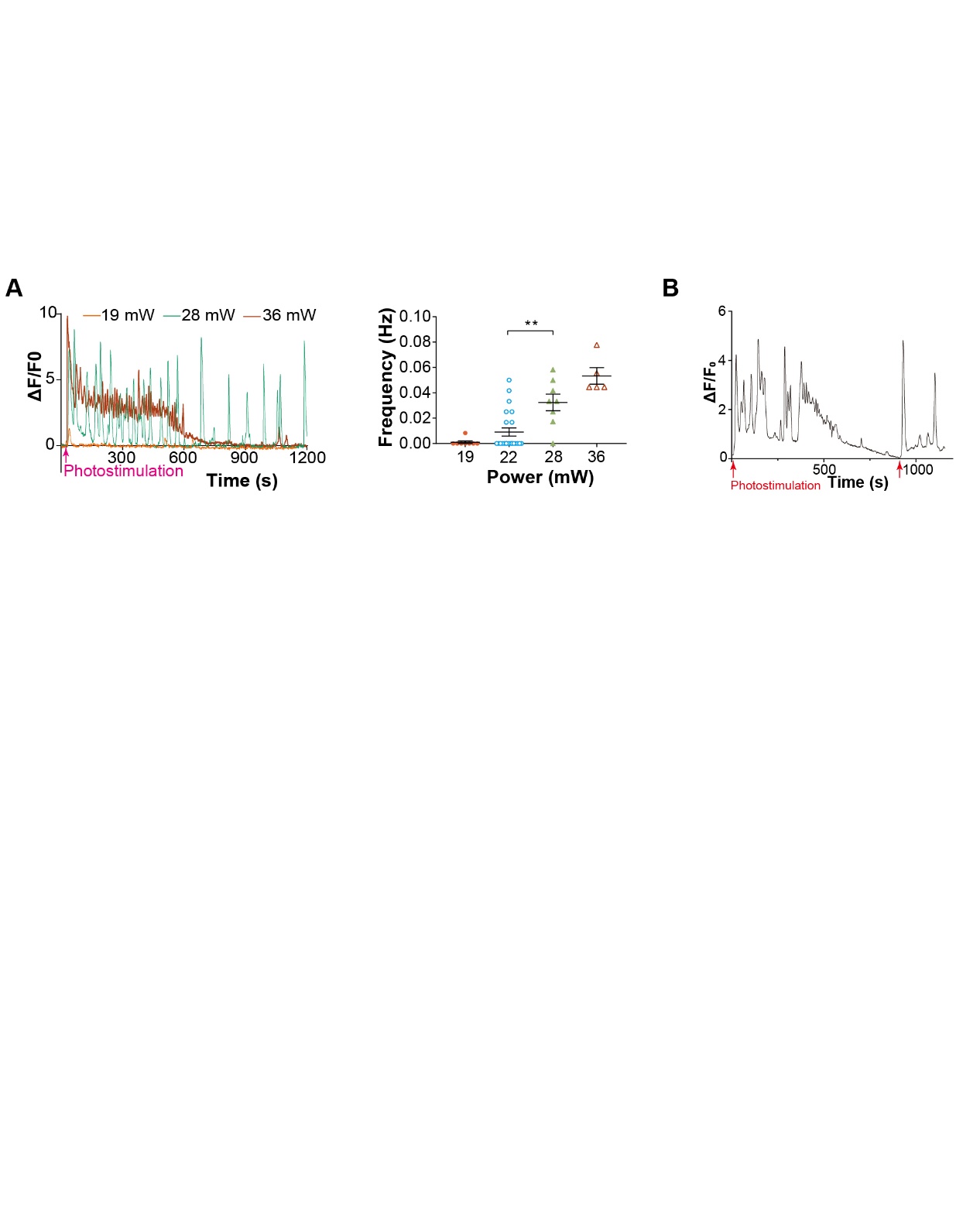


**Supplementary figure 9 Generation of hotspot Ca^2+^ waves by laser. A.** The Ca^2+^ wave oscillations excited by the femtosecond laser at different powers. Left: the amplitude of Ca^2+^ waves passing by an ISC excited by the laser. Right: the average frequency of Ca^2+^ wave generation. **B.** The distribution of Ca^2+^ waves excited by femtosecond laser for two times. Arrows: the laser activation. * *P* < 0.05. ** *P* < 0.01. The cochlea used in Supplementary fig. 9 were all from rats.

**
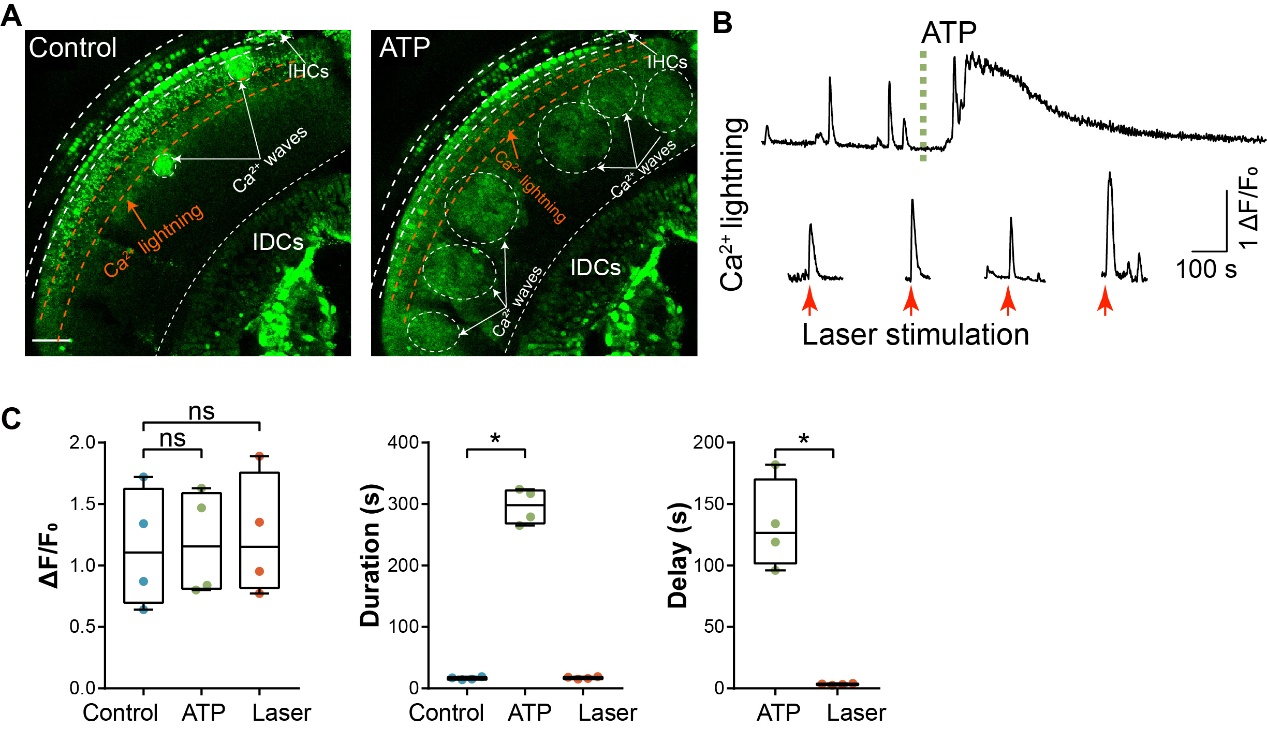
**

**Supplementary figure 10 The Ca^2+^ lightning triggered by ATP treatment.** **A**. Fluorescence images of spontaneous Ca^2+^ lightning (left) and Ca^2+^ lightning triggered by 100 μM ATP (right). Bar: 50 μm. **B**. Amplitude curves of Ca^2+^ lightning triggered by ATP or laser. **C**. Comparison of amplitude, duration, and delay respond of Ca^2+^ lightning triggered by ATP or laser (*n* = 4 cochlea from rats at P6). * *P* < 0.05.

**
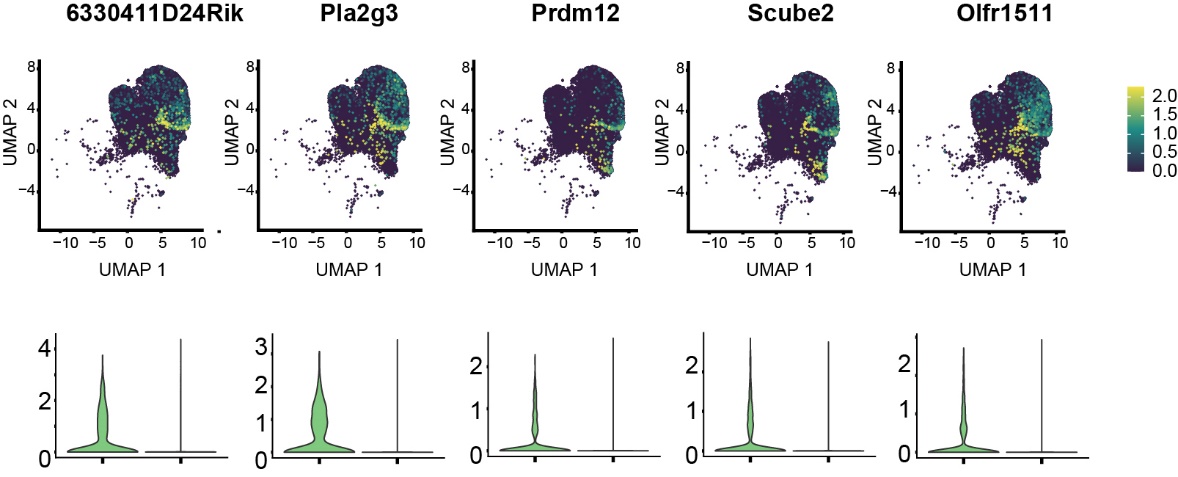
**

**Supplementary figure 11 Violin plots and featured plots of each gene maker of the Ca^2+^ lightning cell cluster** **by comparison with all other cell types.** The cochlea used in Supplementary fig. 11 were all from mice.

**6330411D24rik:** Is expressed in ovary. [provided by Alliance of Genome Resources, Apr 2022]

**Pla2g3：**This gene encodes a protein that belongs to the secreted phospholipase A2 family, whose members include the bee venom enzyme. The encoded enzyme functions in lipid metabolism and catalyzes the calcium-dependent hydrolysis of the sn-2 acyl bond of phospholipids to release arachidonic acid and Lys-phospholipids. This enzyme acts as a negative regulator of ciliogenesis, and may play a role in cancer development by stimulating tumor cell growth and angiogenesis. This gene is associated with oxidative stress, and polymorphisms in this gene are linked to risk for Alzheimer's disease. [provided by RefSeq, Apr 2014]

**Prdm12:** This gene encodes a transcriptional regulator of sensory neuronal specification that plays a critical role in pain perception. The encoded protein contains an N-terminal PRDI-BF1 and RIZ homology (PR) domain, a SET domain, and three C-terminal C2H2 zinc finger DNA-binding domains. Naturally occurring mutations in this gene are associated with congenital insensitivity to pain (CIP), and hereditary sensory and autonomic neuropathies (HSAN's) affecting peripheral sensory and autonomic neurons. Deregulation of this gene is associated with solid cancers and hematological malignancies including chronic myeloid leukemia. [provided by RefSeq, Mar 2017]

**Scube2:** Predicted to enable calcium ion binding activity; hedgehog family protein binding activity; and lipid binding activity. Predicted to be involved in signal transduction. Predicted to act upstream of or within several processes, including positive regulation of chondrocyte proliferation; positive regulation of osteoblast differentiation; and positive regulation of smoothened signaling pathway. Predicted to be located in extracellular region. Predicted to be active in cell surface and extracellular space. [provided by Alliance of Genome Resources, Apr 2022]

**Olfr1511:** Olfactory receptors interact with odorant molecules in the nose, to initiate a neuronal response that triggers the perception of a smell. The olfactory receptor proteins are members of a large family of G-protein-coupled receptors (GPCR) arising from single coding-exon genes. Olfactory receptors share a 7-transmembrane domain structure with many neurotransmitter and hormone receptors and are responsible for the recognition and G protein-mediated transduction of odorant signals. The olfactory receptor gene family is the largest in the genome. The nomenclature assigned to the olfactory receptor genes and proteins for this organism is independent of other organisms. [provided by RefSeq, Jul 2008]


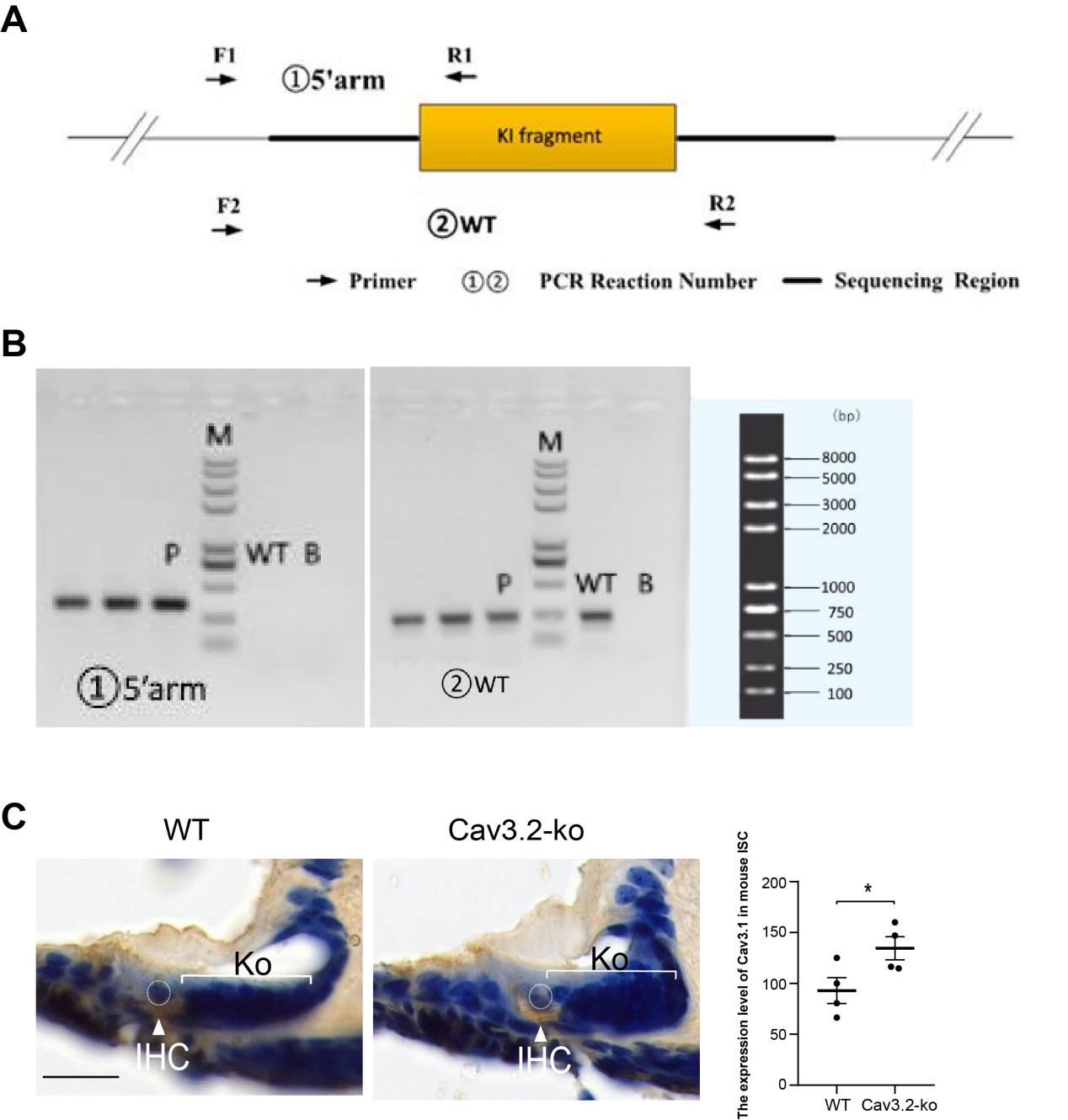


**Supplementary figure 12**. **A**. Strategy of Genotyping. **B**. Gel Image & Conclusion. ①5’arm: 5′- GTCAATGACTTCGCAGACCAG-3′and 5′-CTGACTTCATCAGAGGTGGCATC-3′, which generated a 348-bp band; ②WT: 5′- GTCAATGACTTCGCAGACCAG-3′and 5′- ACGTTCACTTACAGTCTGGTCCC -3′, which generated a 229-bp band. Wild type: ①PCR reaction obtains none band; ②PCR reaction obtains a WT band. Heterozygote: ①PCR reaction obtains a Targeted band; ②PCR reaction obtains a WT band. Homozygote: ①PCR reaction obtains a Targeted band; ②PCR reaction obtains none band. Note: P: Positive control; WT: Wildtype control; B: Blank control (ddH2O); M: DNA Ladder. **C**. Expression analysis of Cav3.1 in cochlear sections from P7 CAV3.2- KO and WT mice. (n = 4 cochleae both for two groups). Scale bar: 20 μm. * p < 0.05; Mann-Whitney test.

**
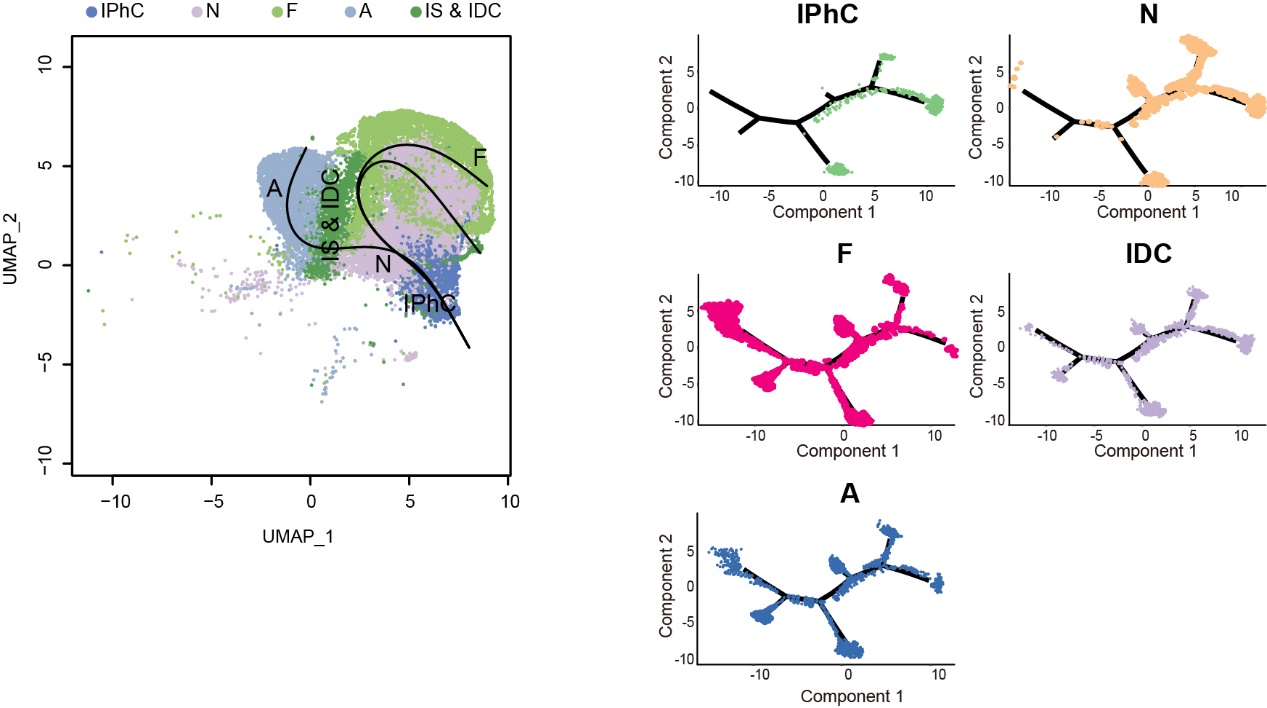
**

**Supplementary figure 13 The trajectory analysis of all cell clusters in Ko and the developmental trajectory each cluster.** The cochlea used in Supplementary fig.13 were all from mice.


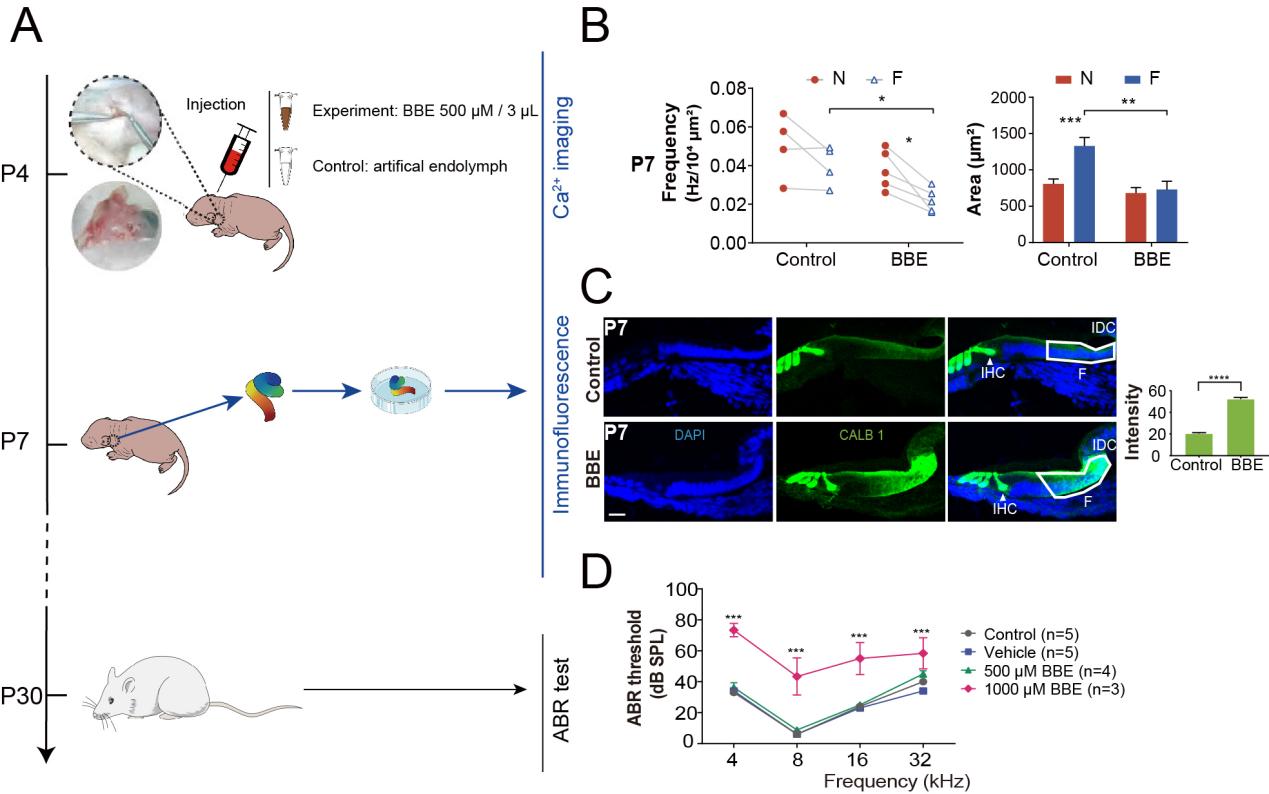


**Supplementary Figure 14 Specific injection of BBE suppresses hearing of rats. A.** Experimental procedures: rat pups were microinjected with BBE into cochleae at P4 and the cochleae were examined at P7. Some injected rats were raised to P30 for ABR threshold testing. Insert: verification of the injection into cochleae by Fast green (concentration: 5%). **B.** The generation frequency and propagation area of spontaneous Ca^2+^ waves at P7 in N and F zones respectively after injecting BBE or artificial endolymph at P4. **C.** IF microscopy of *Calb1* in Ko at P7. Bar: 20 μm. **D.** ABR threshold tested at P30 in rats injected with BBE (500 or 1000 μM) or artificial endolymph (vehicle) at P4, and the control (without any injection) group. *** *p* < 0.001 by post-hoc test (Tukey test) following two-way ANOVA. The cochlea used in Supplementary fig. 14 were all from rats.

**
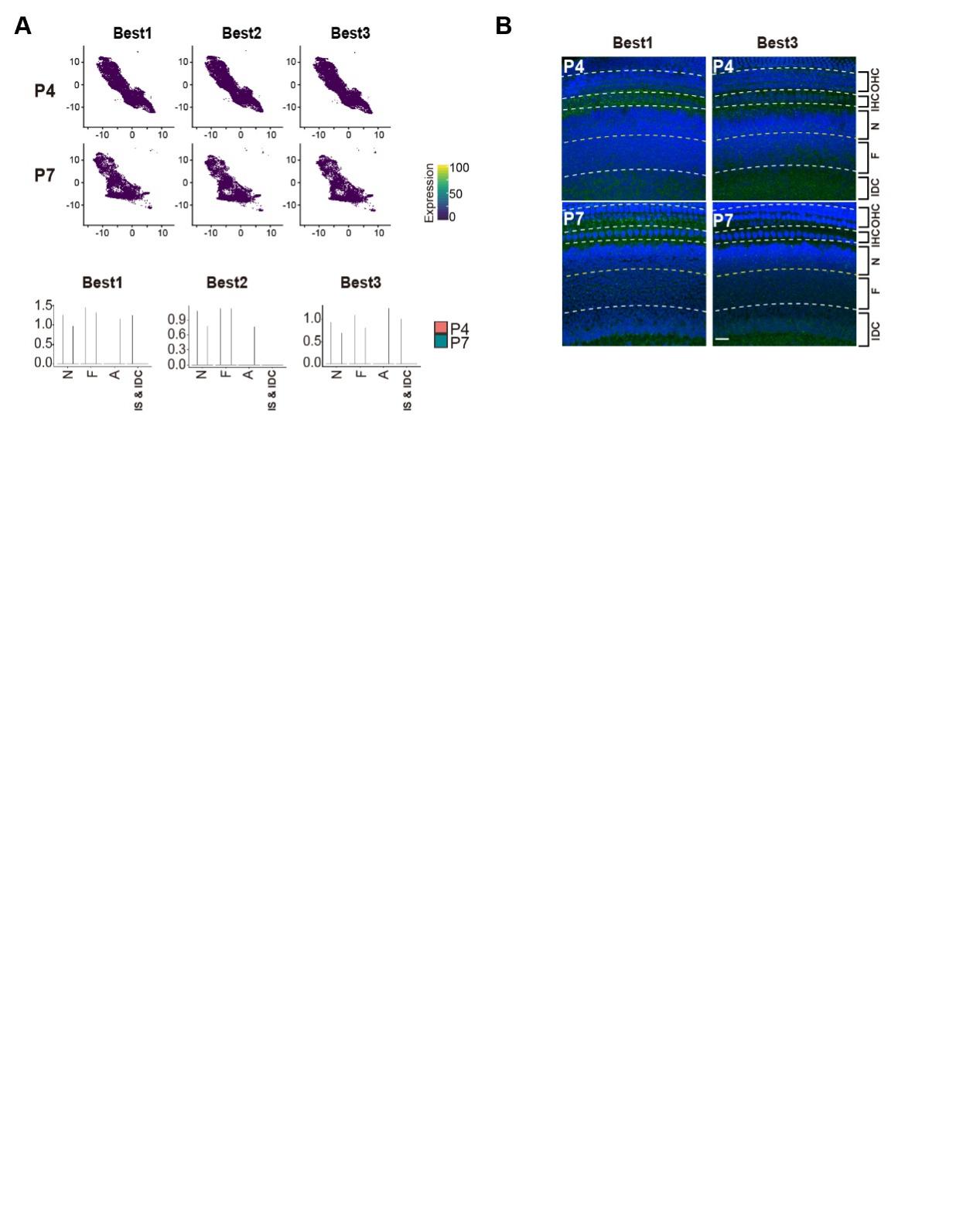
**

**Supplementary figure 15 The** **expression of the Best family in cochleae of rats.** BEST channels form one family of Ca^2+^ dependent Cl^-^ channels. **A.** The violin and featured plots of Best 1-3 in cochleae. **B.** The immunofluorescence microscopy of Best 1 and 3 at P4 and P7 respectively.
